# The bidirectional regulatory mechanism of gut microbiota metabolites on myocardial injury in heart failure from the perspective of the gut-heart axis: a review

**DOI:** 10.3389/fmicb.2025.1710051

**Published:** 2025-11-06

**Authors:** Siyi Guo, Wenhui Zhang, Xiaoxue Cui, Bao Yin

**Affiliations:** 1 Department of Cardiovascular, Zibo Hospital of Traditional Chinese Medicine, Zibo, China; 2 Graduate School, Heilongjiang University of Traditional Chinese Medicine, Harbin, China; 3 First Clinical Medical School, Shandong University of Traditional Chinese Medicine, Jinan, China

**Keywords:** gut-heart axis, heart failure, gut microbiota metabolites, mechanism of action, intervention measures

## Abstract

Dysregulation of gut microbiota-derived metabolites is closely associated with heart failure (HF). However, current research lacks a comprehensive integration of the gut-heart axis regulatory mechanisms, especially regarding an in-depth analysis of the dual roles of key metabolites. This review systematically examines recent advances in the regulation of HF by gut microbiota metabolites, focusing on their bidirectional regulatory mechanisms. Key findings show that HF patients exhibit specific microbial community changes, intestinal barrier damage, and microbiota aging. Toxic metabolites [e.g., trimethylamine N-oxide (TMAO), phenylacetylglutamine (PAGln), and lipopolysaccharide (LPS)] exacerbate HF through mechanisms such as inflammatory activation, oxidative stress, and fibrosis promotion. In contrast, protective metabolites [e.g., short-chain fatty acids (SCFAs), bile acid (BA), hydrogen sulfide (H₂S), and indole derivatives] offer compensatory protection through opposing pathways, including anti-inflammatory effects, antioxidant activity, and maintenance of metabolic homeostasis. Some metabolites demonstrate temporal bidirectional regulation within the same pathological process, with their dual roles dynamically modulated by factors such as dose, timing, host status, and disease stage. Future research should prioritize investigating the metabolite-host interaction network, developing precision intervention strategies, and facilitating the clinical translation of gut-heart axis insights for the precise prevention and treatment of HF.

## 1 Introduction

HF, the terminal stage of several cardiovascular diseases, affects over 64 million individuals globally, despite a decline in the incidence growth rate due to medical advancements (Savarese et al., 2023). Significant regional disparities exist, particularly between developed and developing nations, in terms of HF incidence and treatment. According to Heart Failure Association statistics, among 42 European countries, the median HF prevalence is 17.2 cases per 1,000 individuals, with an annual incidence of 3.2 cases per 1,000 individuals (Beghini et al., 2025). In the Americas, adult HF prevalence ranges from 1.9 to 2.6%, with higher rates in elderly populations, and projections suggest it could reach 8.5% in individuals aged 65–70 years (Bozkurt et al., 2023). Asian countries (e.g., India, China, and Southeast Asian nations) exhibit earlier HF onset, higher incidence rates, and lower treatment levels. Mortality associated with HF remains a critical factor that threatens human lifespan.

Traditional perspectives primarily attribute HF pathogenesis to dysregulation of the neurohumoral-endocrine system and abnormal hemodynamic changes. However, existing clinical treatments show limited efficacy. Neurohormonal therapy is significantly effective in patients with HFrEF but offers minimal impact on HFpEF. Combinations such as angiotensin receptor-neprilysin inhibitors and mineralocorticoid receptor antagonists fail to achieve statistically significant improvements in key HFpEF outcomes. Even standardized combination therapies exhibit a “ceiling effect” in efficacy, with non-negligible risks of hemodynamic deterioration and metabolic/organ toxicity, necessitating urgent attention (Tomasoni et al., 2022). These numerous limitations suggest that current treatment methods cannot fully address the dynamic nature of HF, underscoring the urgent need for new, targeted therapies in clinical practice.

The involvement of gut microbiota dysbiosis in the pathological process of HF, mediated through the bidirectional regulatory mechanisms of the gut-heart axis, has been well-documented. The gut microbiota is an indispensable component of the human body, with the intestinal tract of healthy individuals harboring over 190 genera, at least 500 species, and more than 100 trillion bacteria (Adak and Khan, 2019). Among these, Bacteroidetes and Firmicutes dominate the gut microbiota. The various bacterial communities and their metabolites collaborate to perform functions such as nutrient metabolism, immune regulation, and barrier maintenance, and interact with other systems via pathways like the gut-brain axis, gut-liver axis, and gut-heart axis. The gut microbiota is often referred to as the human “second genome,” and its homeostasis is critical for host health. However, previous research in this area has been inadequate or lacking in depth. With advancements in microbiomics, metabolomics, and immune analysis technologies, previously overlooked aspects have come to light, leading to an explosion of new findings and perspectives. These insights offer fresh approaches for the prevention and treatment of HF.

Our review primarily searched core databases including Google Scholar and PubMed, spanning from January 2015 to September 2025, with supplementary references from earlier literature. The search strategy included a combination of “subject terms + free terms” to cover gut microbiota metabolites (e.g., SCFAs, TMAO), myocardial injury (e.g., HF, ventricular remodeling), and mechanisms (e.g., inflammation, oxidative stress, signaling pathways), using Boolean operators (AND/OR).

Most previous studies exhibit three key limitations: (1) insufficient depth and breadth in mechanistic and target exploration, with unsystematic examination of the potential of metabolites, (2) incomplete evidence systems, marked by an over-reliance on preclinical data and a lack of clinical validation, and (3) weak dialectical analysis, particularly in the dynamic evaluation of the dual effects of metabolites across pathophysiological contexts. Based on the methodology of systematic review, this study adopts a multi-dimensional evidence integration strategy. At the mechanism level, this study focuses on the molecular interaction network between gut microbiota metabolites and HF, with a detailed analysis of regulatory mechanisms involving signaling pathways (e.g., NF-κB and NLRP3) and verification of key targets. At the evidence level, both basic experimental data (cell models and animal model verification) and clinical observation data (cohort studies and RCTs to confirm clinical relevance) are integrated to construct a “bench-to-bedside” translational evidence chain. At the dialectical analysis level, the dual actions of metabolites are evaluated, and the biological plausibility of their “bidirectional regulation” is revealed through dimensions such as dose-effect relationships and dependence on the pathological microenvironment. Finally, this article identifies current research gaps and challenges and offers prospects for future investigations.

## 2 Characteristics and physiological basis of gut microbiota dysbiosis induced by HF

### 2.1 HF-specific alterations in gut microbiota

In the pathological process of HF, gut microbiota dysbiosis represents a critical “environment-host interaction interface,” characterized by a three-dimensional correlation involving phylum-level dysregulation, depletion of protective bacterial genera, and proliferation of pathogenic bacteria. This correlation not only reflects structural alterations in the gut microbiota but also directly contributes to HF progression through microbiota-host co-metabolic interactions.

Clinical studies confirm significant phylum-level remodeling in the gut microbiota of HF patients. Compared to healthy individuals, those with CHF exhibit reduced abundances of Firmicutes and Bacteroidetes, along with elevated levels of Proteobacteria and Actinobacteria (Sun et al., 2022). The decline in Bacteroidetes impairs intestinal barrier function and exacerbates systemic inflammation, while the reduction in Firmicutes leads to an accumulation of TMAO precursors, which promote cardiac injury. The concurrent reduction of both phyla disrupts energy metabolism, accelerating HF progression (Paraskevaidis et al., 2023). In contrast, the proliferation of Proteobacteria and Actinobacteria intensifies inflammation, promotes TMAO production, compromises the intestinal barrier, and induces metabolic disorders, collectively worsening HF (Cienkowski et al., 2024).

Interestingly, phylum-level changes may obscure species-specific adaptive alterations. Despite the overall decline in Bacteroidetes abundance in HF patients, certain members (e.g., *Bacteroides dorei*, *Alistipes putredinis*, *Eubacterium eligens*) and Firmicutes taxa (e.g., Lachnospiraceae, *Dorea formicigenerans*) show proliferative advantages in the HF environment (Dai et al., 2023). These seemingly contradictory observations likely reflect compensatory responses within the gut microbiota. This “intra-phylum heterogeneity” suggests that microbiota changes in HF are not merely quantitative but also involve adaptive reprogramming of bacterial species.

From a more nuanced perspective, the depletion of protective genera and the proliferation of pathogenic bacteria are specific manifestations of phylum-level dysregulation. The loss of protective genera (e.g., Parabacteroides, *Faecalibacterium prausnitzii*) impairs their ability to metabolize dietary fiber into secondary BAs or SCFAs, leading to dysfunction in intestinal barrier repair, inflammation suppression, and lipid metabolism regulation (Chen et al., 2023). Meanwhile, the expansion of pathogenic bacteria (e.g., *Escherichia coli*, Collinsella) due to the growth of Proteobacteria and Actinobacteria contributes to vascular endothelial dysfunction, aggravated myocardial fibrosis, impaired bile acid reabsorption, and increased pro-inflammatory factor secretion, thereby directly exacerbating myocardial injury (Rizzatti et al., 2017). Notably, this dynamic interplay between protective and pathogenic bacteria is not isolated but forms a positive feedback loop within the microbiota-host co-metabolic network.

### 2.2 Intestinal barrier damage and the mechanism of “leaky gut”

HF drives intestinal barrier injury through a unique “ischemia-congestion-neurohormonal triad,” forming the pathological basis for bacterial metabolite translocation and myocardial toxic injury. Intestinal ischemia in HF arises from two mechanisms: reduced cardiac output-induced intestinal hypoperfusion and hypercoagulability-mediated thromboembolism. Specifically, HF reduces cardiac output, directly decreasing blood flow to peripheral tissues and organs, leading to intestinal ischemia. Additionally, HF patients exhibit hypercoagulable blood with elevated left atrial pressure, which predisposes to left atrial thrombus formation and mesenteric artery embolism—the most severe form of intestinal ischemia (Bala et al., 2022). Studies confirm that chronic HF reduces mesenteric artery blood flow by over 30%, with ischemia severity strongly correlated with the deterioration of cardiac function (Sandek et al., 2014).

Persistent ischemia directly induces mitochondrial dysfunction in intestinal epithelial cells, exacerbating oxidative stress and impairing ATP synthesis. This activates matrix metalloproteinase-9, a proteolytic enzyme involved in tissue remodeling and inflammation via extracellular matrix degradation (Barron et al., 2024). Ischemia also disrupts tight junction (TJ) protein complexes, characterized by increased disassembly/reduced synthesis of TJs and enhanced degradation of Occludin and ZO-1 (a cytoskeletal scaffolding protein that stabilizes the TJ structure through the Occludin-cytoskeleton linkage) (Kuo et al., 2022). This process ultimately increases paracellular permeability. Notably, HF patients exhibit reduced intestinal mucus layer thickness compared to healthy individuals. MUC2, a core barrier component and key regulatory target, is suppressed under dual inhibition by ischemia-activated HIF-1*α* and TNF-α-mediated NF-κB signaling, which decreases MUC2 promoter activity and hinders its transcription/expression in intestinal epithelial cells (Liu et al., 2023). This weakening of the physical barrier promotes microbial translocation.

Similar to ischemia, HF-induced intestinal congestion disrupts the barrier through hypoxia, structural damage, and immune imbalance, likely due to microcirculatory stagnation from erythrocyte aggregation and leukocyte adhesion (Chen et al., 2021). During this process, neutrophil extracellular trap (NET) release activates mitochondrial autophagy imbalance, causing ferroptosis and excessive ROS generation (Chu et al., 2023). NOX activation further exacerbates oxidative stress, disrupts TJs, and directly impairs barrier integrity. HF also induces portal hypertension through hepatic congestion, elevating mesenteric venous and capillary hydrostatic pressure, increasing fluid extravasation into the intestinal interstitium, and causing intestinal wall edema and widened intercellular spaces (Boccatonda et al., 2024). Congestion induces vasodilation, slowed blood flow, reduced vasoactive substances (e.g., NO), and increased injury factors, worsening mucosal hypoxia and barrier damage (Ikeda et al., 2018). Additionally, congestion alters gut microbiota composition, promoting anaerobic pathogen overgrowth (e.g., Clostridium) and reducing the abundance of aerobic beneficial bacteria (e.g., Lactobacillus). This shift decreases the secretion of beneficial products (e.g., SCFAs) and impairs the metabolism and utilization of intermediate metabolites (e.g., BAs) (Hu et al., 2022).

In HF, reduced effective circulating blood volume triggers renal hypoperfusion, while central sympathetic overactivation forms a positive feedback loop, leading to sustained RAAS activation. This significantly elevates Angiotensin II (Ang II) levels, a key RAAS mediator, which induces intestinal epithelial injury through four main pathways: (1) Ang II selectively inhibits P-glycoprotein (Pgp) function and membrane expression in intestinal epithelial cells via AT1 receptor-mediated PI3K/Akt-p38 MAPK signaling. Impaired Pgp, an ATP-dependent transmembrane transporter, directly increases intestinal endotoxin absorption (Kumar et al., 2020). (2) Ang II induces mitochondrial membrane potential depolarization and lipid metabolic reprogramming (increased synthesis of saturated fatty acids and decreased synthesis of unsaturated fatty acids), disrupting mitochondrial ATP synthesis, compromising cellular energy homeostasis, and reducing intestinal epithelial repair capacity (Toth et al., 2024). (3) Ang II upregulates AT2 receptor-dependent GATA-6 transcription factor and Bax protein gene transcription, promoting apoptosis (Sun et al., 2012). (4) Ang II upregulates AT1 receptor-induced COX-2 expression, promoting prostaglandin synthesis and forming an AT1-COX-2-PGs pro-inflammatory axis. This axis synergizes with LPS from gut microbiota translocation, elevating pro-inflammatory cytokine levels and amplifying inflammation (Tani et al., 2008).

Gut bacterial translocation is a core pathogenic mechanism driving HF progression, perpetuating the disease through a vicious cycle of “barrier disruption, metabolic dysregulation, and inflammatory amplification.” In HF, reduced cardiac output and venous congestion induce intestinal hypoperfusion, leading to decreased expression of intestinal epithelial tight junction proteins and mucosal atrophy, creating a “leaky gut” state (Chen et al., 2023). Concurrently, pathogenic bacteria (e.g., Proteobacteria overgrowth) release metabolites such as LPS, which translocate into the systemic circulation via the compromised barrier. LPS triggers systemic inflammation through the TLR4-NF-κB pathway, promoting the release of pro-inflammatory cytokines and directly exacerbating myocardial apoptosis, fibrosis, and microvascular obstruction, thus forming an “injury-translocation-inflammation-worsened injury” cascade (Snelson et al., 2025). Barrier disruption further depletes beneficial bacteria (e.g., SCFA-producing species), reducing the production of protective metabolites like butyrate. SCFAs enhance intestinal barrier repair via histone deacetylase inhibition and suppress inflammation through anti-inflammatory pathways. Their deficiency perpetuates barrier dysfunction, amplifying LPS translocation and inflammatory responses. In elderly patients, age-related intestinal barrier decline and systemic inflammation establish an “aging-gut-heart” axis, accelerating HF progression. Clinical interventions support this mechanism: antibiotic-mediated gut microbiota depletion reduced LPS translocation, alleviated inflammation, and improved cardiac function. GLP-2 administration in myocardial I/R models promoted L cell secretion, enhanced tight junction protein expression, and inhibited TLR4 signaling, thereby repairing the intestinal barrier and blocking bacterial translocation. These findings confirm that targeting gut bacterial translocation and its associated pathways offers novel precision treatment strategies for HF (Zhao et al., 2023).

Notably, excessive use of loop diuretics (e.g., furosemide) in HF treatment stimulates RAAS activation and induces intestinal dehydration, significantly reducing the mucus layer thickness and compromising its protective function (Eid et al., 2021). This disruption of the physical barrier, coupled with Ang II-mediated impairment of the chemical barrier, creates an imbalance between intestinal epithelial injury and regeneration, setting up a vicious cycle.

### 2.3 Gut microbiota aging

To characterize age-related functional alterations in the gut microbiota, the concept of “gut microbiota aging” has been proposed, offering a novel framework to explain age-associated physiological decline. The core of this concept lies in identifying the degenerative functional traits (rather than just abundance shifts) of the microbiota during host aging. As the terminal stage of cardiovascular diseases, HF primarily affects elderly populations. Previous studies have confirmed a synergistic relationship between cardiac structural/functional aging and gut microbiota aging, with HF accelerating the latter. The “gut microbial age” metric, which quantifies the deviation between actual physiological age and microbiota age by integrating microbiome features with host metabolic data, has been shown to independently predict adverse cardiovascular outcomes (Wang et al., 2024). Specifically, among individuals over 60, those with a younger microbiota age have a lower cardiovascular risk despite metabolic disorders, while those with an older microbiota age face higher risk even when metabolically healthy.

A study on male Wistar rats further supports the age dependency of microbiota function, showing that during middle-to-old age, the Rhodococcus genus may act as a central hub linking organ functional decline to microbiota aging by regulating key metabolic pathways (e.g., essential amino acid degradation and protective substance synthesis) (Yang et al., 2021). Human studies have identified core dysbiosis features in aging, including compositional imbalance within the Bacteroidetes phylum, reduced Prevotella abundance, and abnormal proliferation of Clostridiales (O'Toole and Jeffery, 2015). These structural changes drive enhanced toxicity of protein fermentation products, impaired carbohydrate utilization, and chronic depletion of host nutritional reserves.

To assess stage-specific microbiota changes in HF progression, Spehlmann et al. found that in the early compensatory stage, gut *α*-diversity begins to decline along with reductions in SCFA-synthesizing species, despite no significant changes in overall species abundance (Spehlmann et al., 2022). In the decompensated stage, marked species remodeling occurs: increases in Bacteroidetes/Firmicutes genera, coupled with decreases in SCFA-producing bacteria and genera from Firmicutes, Deferribacteres, and Proteobacteria. Cohort studies confirm elevated Escherichia/Shigella abundance at this stage (Hayashi et al., 2018).

Aging cardiomyocytes, under the combined effects of pump failure, neuroendocrine overactivation, metabolic remodeling, and oxidative stress, further exacerbate gut microbiota *α*-diversity loss and metabolite imbalance via mechanisms such as aggravated insulin resistance, advanced glycation end-product accumulation, and metabolic acidosis. Regarding microbiota functional dynamics in HF, Modrego et al. reported stage-dependent evolutionary traits: early-to-mid HF stages show progressive SCFA synthesis decline and rising IL-6/TNF-α levels, while late HF stages exhibit near-depleted SCFAs and cytokine storms triggering multi-organ dysfunction (Modrego et al., 2023). Han et al. further demonstrated that microbiota aging in HF mice induces glucolipid metabolic disorders, systemic metabolic shifts, and impaired protective capacity, while exacerbating intestinal barrier damage. This allows LPS translocation, activates inflammation, downregulates superoxide dismutase/glutathione activity, increases malondialdehyde accumulation, and aggravates oxidative stress (Xu et al., 2024).

**Figure 1:**
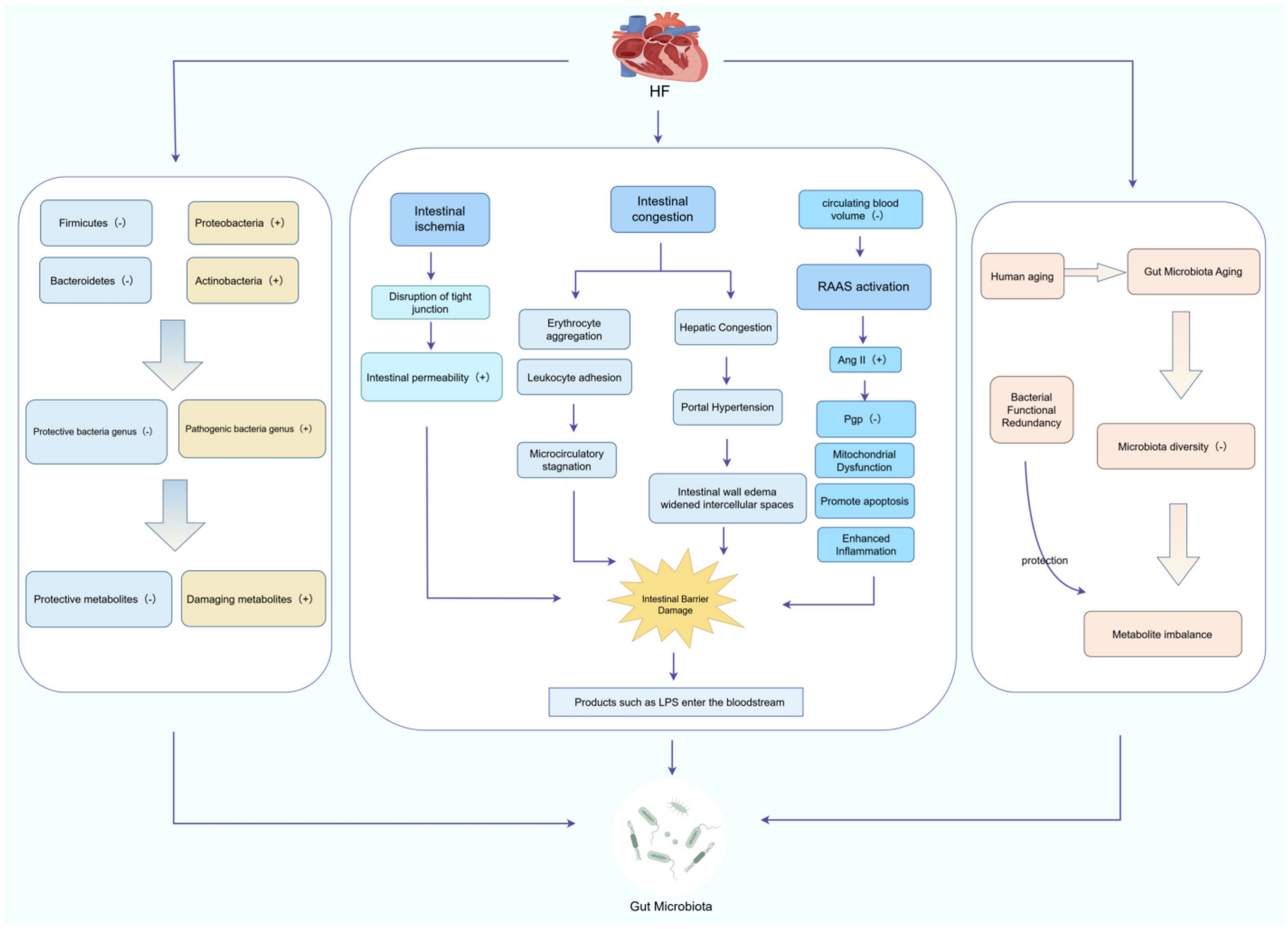
Mechanism of HF-induced gut microbiota damage. HF causes gut microbiota damage through a three-dimensional mechanism that includes specific alterations in gut microbiota, intestinal barrier damage, and gut microbiota aging. The (+) and (−) symbols in the figure represent increases and decreases, respectively. This figure was created using FigDraw software.

Notably, while some studies report reduced SCFA synthesis in the elderly, Badal et al. systematically analyzed age-related microbiota reconstruction and proposed that SCFA abundance is higher in the “oldest-old” (≥80 years), suggesting adaptive co-metabolic mechanisms (Badal et al., 2020). We hypothesize that this compensation may stem from functional redundancy in previously unrecognized microbiota components. Although this mechanism partially alleviates metabolic pressure, it may weaken the microbiota-host protective effects, leading to a “functional compromise-risk accumulation” vicious cycle. Finally, whether long-term HF treatments (e.g., *β*-blockers, diuretics, SGLT2 inhibitors) indirectly affect microbiota drug-metabolizing gene expression through altered intestinal electrolyte balance or immune microenvironment remains to be explored (Figure 1).

## 3 Core mechanisms of HF mediated by gut microbiota metabolites

**Figure 2:**
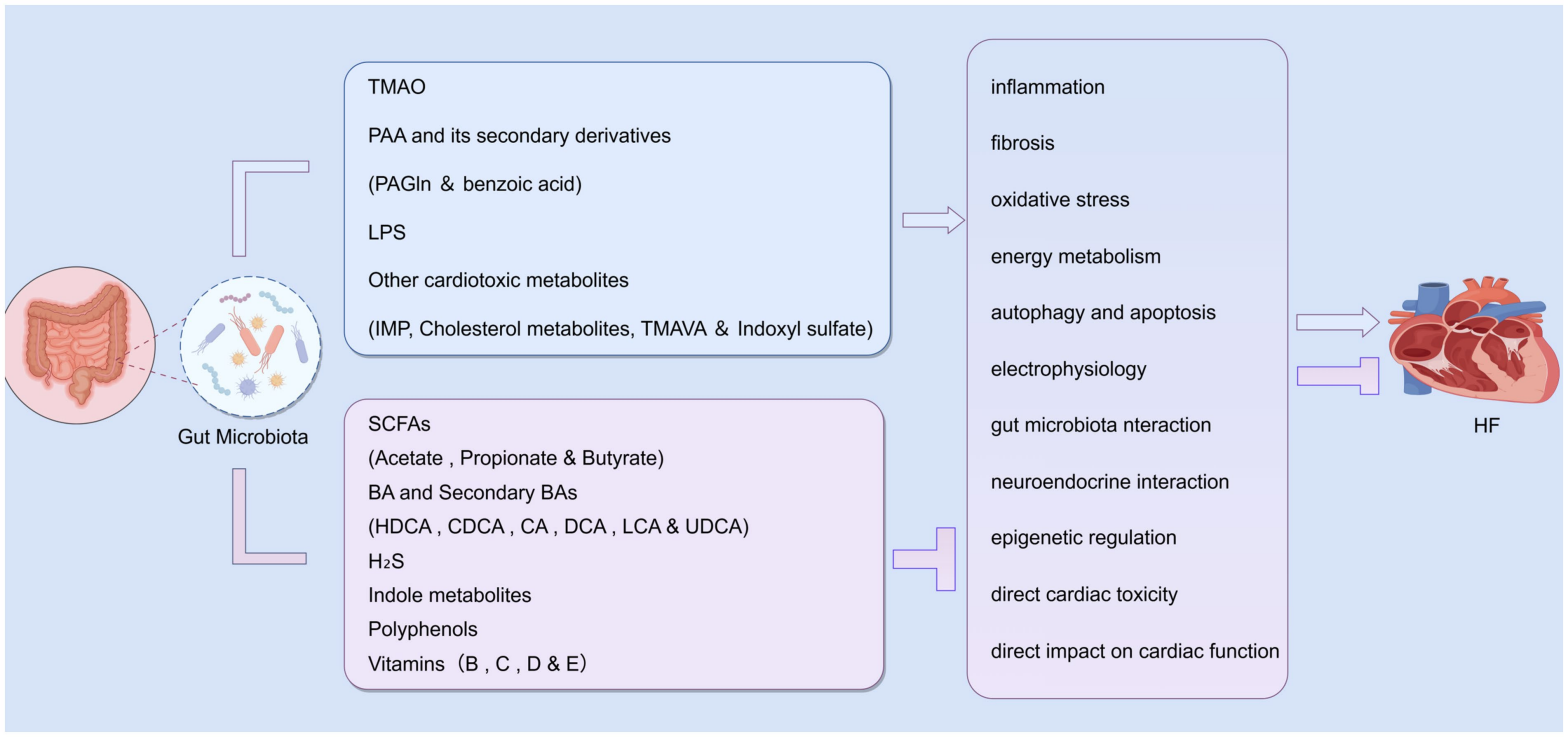
Dual mechanisms of gut microbiota action on HF. Among these metabolites, TMAO, PAA and its secondary derivatives, LPS, and other cardiotoxic metabolites exacerbate HF through mechanisms such as inflammation, fibrosis, and oxidative stress. Conversely, SCFAs, primary/secondary BAs, H₂S, indole derivatives, polyphenols, vitamins, and other beneficial compounds inhibit these pathogenic pathways to alleviate HF. This figure was created using FigDraw software.

We systematically investigated the multifaceted roles and clinical significance of gut microbiota-derived metabolites in the pathogenesis of HF. This investigation identified two major categories of metabolic modulators: deleterious metabolites that exacerbate disease progression, and beneficial metabolites that exert protective effects. These metabolites exhibit dual regulatory functions in HF pathogenesis, either accelerating or retarding disease progression through a variety of mechanisms. Key pathways include modulation of inflammatory responses, oxidative stress, metabolic dysregulation, and neuro-immune interactions. Importantly, their plasma concentrations serve as valuable biomarkers for risk stratification and prognostic assessment in clinical settings. In the pathophysiological conditions of HF, a characteristic metabolic imbalance emerges—characterized by elevated levels of deleterious metabolites and reduced concentrations of protective metabolites. This imbalance exacerbates pathological effects while diminishing the body’s endogenous protective capacities. Notably, certain metabolites may exhibit context-dependent functional polarity, where even the same metabolite can produce opposing effects depending on experimental conditions, dosage regimens, or disease stages. These paradoxical phenomena highlight the intrinsic complexity of human physiological regulation in real-world clinical scenarios (Figure 2).

### 3.1 Myocardial injury driven by injurious metabolites

#### 3.1.1 TMAO

TMAO is the end product of TMA, which is produced when intestinal microorganisms metabolize choline, carnitine, and other nutrients, followed by hepatic oxidation via flavin monooxygenase 3. As an independent predictor, TMAO is directly associated with adverse clinical outcomes in HF patients, including all-cause hospitalization and mortality. TMAO contributes to disease progression through multiple mechanisms: on one hand, it promotes inflammation, enhances oxidative stress, exacerbates myocardial necrosis, and drives metabolic remodeling, ultimately inducing myocardial injury. On the other hand, under specific pathophysiological conditions (e.g., low-dose exposure or acute stress), TMAO activates endoplasmic reticulum (ER) stress-protective pathways or upregulates antioxidant enzyme expression, demonstrating a potential compensatory effect on myocardial tissue. This “pro-injury and compensatory protection” duality underlies TMAO’s paradoxical roles in HF.

##### 3.1.1.1 Pro-inflammatory effect

TMAO directly participates in cardiac structural remodeling and cardiomyocyte injury by mediating inflammatory responses. Early studies confirm that TMAO activates the MAPK and NF-κB signaling pathways, initiating inflammatory cascades (Seldin et al., 2016). In macrophage polarization, TMAO upregulates METTL3 (an m6A “writer” enzyme), which catalyzes m6A modifications on IRAK-M mRNA. This modification facilitates YTHDF2-mediated recognition and degradation of target mRNA, reducing IRAK-M protein levels. As IRAK-M negatively regulates NF-κB, its depletion relieves NF-κB inhibition, driving macrophages toward a pro-inflammatory (M1) phenotype and promoting cytokine release. This establishes a novel TMAO-m6A/METTL3/YTHDF2 axis that regulates macrophage function and valvular inflammation (Wen et al., 2025). Furthermore, TMAO enhances TNF-*α*-mediated NF-κB/MAPK pathway activity, upregulating pro-inflammatory factors and chemokines, which recruit immune cells and exacerbate inflammation (Stefania et al., 2024). In myocardial injury models, TMAO activates NF-κB to upregulate IL-8, promoting neutrophil infiltration and cytokine release. Simultaneously, it enhances platelet activity, induces foam cell formation, and thereby initiates a vicious cycle that aggravates myocardial injury (Li et al., 2019). In adriamycin-induced models, TMAO exacerbates damage via NLRP3 inflammasome activation and oxidative stress (through TLR4/ROS accumulation) (Li et al., 2019). Myocardial vascular inflammation can spread to myocardial cells via inflammatory mediators. Vascular inflammation includes TMAO-induced disruption of phospholipid metabolism via MBOAT2 upregulation, triggering ER stress and NLRP3 activation. This prompts caspase-1-mediated GSDMD cleavage, generating membrane pores and inducing pyroptosis in endothelial cells (Yu et al., 2024). This injury further exacerbates myocardial damage.

It is worth noting that temporal dynamics reveal bidirectional regulation: early (24 h) TMAO inhibits TNF signaling to suppress inflammation, while later (48 h) it activates NOD-like receptors and enhances IL-6 expression (Shanmugham et al., 2023), forming an “early inhibition-late activation” pattern. This temporal regulation likely relates to changes in metabolic enzyme activity. These results demonstrate the “window of action” effect of TMAO and provide important insights for its clinical translation.

##### 3.1.1.2 Pro-fibrotic effects

TMAO drives cardiac fibrosis progression via dose-dependent mechanisms. TGF-*β* superfamily pathway activation occurs: TMAO promotes cardiac fibroblast proliferation, migration, and collagen secretion through the TGF-β/Smad3 pathway in a dose-dependent manner, with pathway activity regulated by the NLRP3 inflammasome (Li et al., 2019). In TAC-induced hypertrophy models, elevated plasma TMAO correlates with myocardial fibrosis severity. Treatment with 5 μM TMAO for 18 h activates TGF-*β*1/SMAD3 signaling, upregulates ANP/β-MHC expression, and exacerbates pathological hypertrophy (Li et al., 2019). Tang et al. further demonstrated that TMAO inhibits TGF-*β*RI ubiquitination, reducing proteasomal degradation, prolonging its half-life, and sustaining Smad2 phosphorylation/fibrotic gene transcription (e.g., *α*-SMA, collagen I) (Yang et al., 2019).

Secondly, UPR involvement: Xiong et al. reported that TMAO triggers dose-dependent unfolded protein response in aortic valve interstitial cells, increasing PERK/IRE-1α phosphorylation and ATF-4/XBP-1 s expression (Xiong et al., 2024). Through these two pathways, which promotes TGF-β1/α-SMA/collagen I synthesis, driving AVIC-to-myofibroblast differentiation and ECM deposition, linking ER stress to fibrotic phenotypic transformation.

Thirdly, inflammatory signaling: In myocardial infarction models, TMAO activates JAK2-STAT3 in cardiac fibroblasts, increasing JAK2/STAT3 phosphorylation and fibronectin/collagen I/III synthesis (Yang et al., 2025). Nian et al. confirmed TMAO induces KRT17 expression (regulating EMT), activates TGF-β/Smad/TLR4/NF-κB pathways, and promotes fibroblast activation, collagen deposition, and inflammation (Nian et al., 2023). Concurrently, it exacerbates hypertrophic injury via integrin-FAK/mTORC1 axis-mediated metabolic reprogramming.

Finally, epigenetic regulation: Yang et al. showed TMAO activates Wnt3a/β-catenin in atrial fibroblasts, inducing β-catenin nuclear translocation and TGF-β1 upregulation, promoting fibroblast-to-myofibroblast transformation (Yang et al., 2022). Another study found TMAO downregulates Piezo1, activating NOX4/ROS oxidative stress, causing abnormal calcium handling and fibrosis, constituting the molecular network of TMAO-induced cardiac fibrosis (Chen et al., 2025).

The above “quadruple network” links cardiac fibroblast proliferation, ER stress response, inflammatory signals and epigenetics, which together constitute the pathological basis of TMAO-induced fibrosis.

##### 3.1.1.3 Oxidative stress

Oxidative stress constitutes a core pathological mechanism driving the onset and progression of HF. At the molecular signaling level, TMAO inhibits ATP-induced intracellular calcium influx and eNOS phosphorylation at Ser1179, reducing NO release, triggering calcium-NO signaling uncoupling, diminishing NO’s ability to scavenge superoxide anions, and exacerbating ROS accumulation (Querio et al., 2022). Additional studies show that TMAO inhibits SIRT1 deacetylase activity, triggering mitochondrial dysfunction and ROS accumulation, which leads to cell cycle arrest and impaired proliferative/migratory capacities (Ke et al., 2018). At the level of mitochondrial metabolic reprogramming, TMAO disrupts pyruvate and fatty acid oxidation, impairs electron transport chain function, reduces ATP synthesis, and induces energy metabolism disorders with concurrent ROS accumulation (Makrecka-Kuka et al., 2017).

Nrf2 serves as a key regulatory node and has demonstrated a dual regulatory effect in existing studies. Mechanistic analyses reveal that TMAO activates the NOX pathway to increase ROS production. Elevated ROS, in turn, activate the Nrf2 transcription factor, upregulating CES1 expression and enzyme activity, thereby forming the NOX/ROS/Nrf2/CES1 signaling axis, which constitutes a self-amplifying oxidative stress cascade (Ge et al., 2023). Another study shows that TMAO suppresses Nrf2 and its downstream antioxidant response elements, leading to increased intracellular ROS generation and reduced SOD activity (Luo et al., 2024). However, notably, TMAO also exerts protective effects by activating the Nrf2 pathway, promoting Nrf2 dissociation from Keap1 and nuclear translocation, thereby inducing antioxidant genes (HO-1, NQO1, CAT) to reduce ROS accumulation and restore antioxidant enzyme activity—underscoring Nrf2’s dual regulatory role in oxidative stress (Zou et al., 2024).

However, in *ex vivo* cardiomyocyte models, TMAO concentrations (100 μM–10 mM) did not significantly alter oxidative stress markers nor enhance the cytotoxicity of known oxidative stress inducers, suggesting it may not serve as a direct mediator of acute cardiac oxidative stress (Querio et al., 2019). Conversely, its precursor TMA, at physiological/pathological concentrations (0.6/1.2 μM), directly impairs macrophage mitochondrial function by reducing ATP production, altering membrane potential, and upregulating mitochondrial genes (mt-ATP6, mt-CO1), thereby triggering mitochondrial stress responses (Bordoni et al., 2025).

##### 3.1.1.4 Metabolic dysregulation

The repair of cardiomyocytes after HF injury requires significant energy maintenance. However, the metabolic imbalance caused by TMAO interferes with energy supply through multiple mechanisms. First, in terms of its effect on mitochondria, TMAO upregulates HSP90*β* at the mitochondrial level, disrupting proteostasis and causing the accumulation of ubiquitinated proteins. This induces mitochondrial depolarization, ROS overproduction, mPTP opening, and lipid metabolic dysregulation (Guo et al., 2025). Metabolic toxicity runs through different stages of TMAO injury: acute exposure reduces mitochondrial pyruvate dehydrogenase flux, impairing LEAK and OXPHOS respiration. Whereas chronic exposure specifically inhibits palmitoyl-CoA-dependent β-oxidation, creating a dual blockade of substrate utilization that induces energy imbalance (Makrecka-Kuka et al., 2017). The most direct consequence of mitochondrial damage is the lack of energy supply, which directly leads to a decline in myocardial cell function.

In lipid metabolism, TMAO binds DPP4 to inhibit antioxidant enzymes, promoting iron accumulation and lipid peroxidation (Wang et al., 2024). It also suppresses ABCA1 via the Nrf2/ABCA1 pathway, reducing cholesterol efflux and promoting macrophage foam cell lipid accumulation (Makrecka-Kuka et al., 2017). Mechanistic studies in apoE−/− mice reveal that TMAO activates the FXR/SHP axis, specifically repressing Cyp7a1—the rate-limiting enzyme in bile acid synthesis—thereby blocking classical bile acid synthesis and reducing cholesterol excretion. Concurrently, it upregulates bile acid transporters Abcb11/Slc10a1, establishing an FXR/SHP-Cyp7a1 feedback axis that exacerbates lipid dysregulation (Ding et al., 2018). Abnormal lipid metabolism interferes with the normal physiological environment of cardiomyocytes, potentially aggravating normal metabolism, contractile activity, and promoting cell fibrosis and death.

Notably, TMAO’s regulatory effects are context-dependent. In right ventricular HF rats, long-term TMAO intervention induces metabolic reprogramming: it reduces fatty acid oxidation-dependent respiration while enhancing pyruvate metabolic efficiency, enabling an adaptive substrate shift from fatty acids to glucose under hypoxia (Videja et al., 2021). This mechanism, independent of mitochondrial mass changes, optimizes complex I/II activity and regulates carnitine acylcarnitine translocase to enhance substrate switching. In FXR knockout mice, TMAO modulates hepatic bile acid/cholesterol transporter and synthase mRNA expression, reducing bile acid synthesis and cholesterol accumulation (Miyata et al., 2024). These findings suggest that TMAO’s effects on metabolic function are contingent on specific contexts.

##### 3.1.1.5 Direct cardiac and electrophysiological effects

TMAO exhibits concentration- and time-dependent regulation of cardiac function. It induces direct cardiomyocyte toxicity: adult rat ventricular myocytes exposed to 20–100 μM TMAO show a 26–28% reduction in fractional shortening, a 31–47% decrease in maximal shortening/relengthening rates, and a 13–20% prolongation of calcium removal time (Savi et al., 2018). In isolated myocardium, TMAO produces acute inotropic effects—enhancing calcium influx via channel potentiation, increasing myocardial contractility, and stabilizing ion channel integrity to augment excitability and sinoatrial node pacing, thereby transiently maintaining cardiac output (Oakley et al., 2020). Unfortunately, such a short window of potency has not been demonstrated *in vivo*. In sharp contrast to acute exposure, long-term exposure to high concentrations can induce compensatory hypertrophy and structural remodeling, accelerating the progression of HF. In HFpEF models, TMAO impairs diastolic function by inhibiting Piezo1 expression and disrupting mechanotransduction (Chen et al., 2025). Additionally, TMAO dose-dependently suppresses TRPV4-mediated Ca^2+^ influx, blocking the TRPV4-NO pathway to reduce endothelial NO production and compromise vasodilation (Zhang et al., 2024). It also contracts vascular smooth muscle and modulates calcium channels to exacerbate vasoconstriction, increasing cardiac energy expenditure (Restini et al., 2021).

Notably, TMAO’s cardiac effects follow concentration gradients: moderate concentrations (10–100 μM) reduce LVDP and ±dP/dt by inhibiting coronary hemodynamics, while high concentrations (≥300 μM) worsen systolic inhibition and elevate LVEDP (Naghipour et al., 2023), indicating deteriorating pump function. Paradoxically, in chronic hypertension models, low-dose TMAO improves systolic/diastolic function and reduces fibrosis by stabilizing myocardial protein structure (Huc et al., 2018). This dual-effect paradigm underscores the importance of evaluating TMAO’s impact in the context of dose, duration, and pathophysiological background.

TMAO mediates abnormal neural electroactivity regulation via direct activation of the cardiac sympathetic nervous system and indirect modulation of central autonomic pathways. Specifically, TMAO enhances cardiac sympathetic activity through both direct (left stellate ganglion) and indirect (central autonomic) pathways, while concurrently upregulating pro-inflammatory cytokine release. This dual action induces atrial autonomic dysfunction and electrophysiological instability (Yu et al., 2018). This mechanism aligns with findings by Wang et al., where TMAO downregulates P2Y12 receptor expression in paraventricular nucleus microglia, establishing a “TMAO-P2Y12R inhibition-neuroinflammation” positive feedback loop. This loop significantly amplifies renal sympathetic nerve activity and exacerbates sympathetic hyperactivity (Wang et al., 2024), providing a novel mechanism for aging-related cardiac dysfunction.

In metabolic diseases, TMAO’s pathogenic effects are potentiated. In T2DM rat models, TMAO disrupts intercellular signal synchronization by interfering with connexin 43 expression and distribution in cardiomyocytes, resulting in atrial electrical conduction abnormalities and electrophysiological derangements (Jiang et al., 2022). Cellular validation confirms this dual impact of structural remodeling and functional dysregulation: TMAO impairs T-tubule integrity in cardiomyocytes, disrupting calcium transient spatiotemporal synchrony. This manifests as reduced calcium influx rate, decreased sarcoplasmic reticulum Ca^2+^ release/reuptake efficiency, and slowed contraction/relaxation kinetics, directly impairing excitation-contraction coupling (Jin et al., 2020).

Notably, TMAO demonstrates trans-organ pathological synergy. In diabetic mice, it induces hippocampal oxidative stress and PERK-eIF2α-mediated ER stress, potentially disrupting systemic calcium homeostasis to impair cardiomyocyte function (Govindarajulu et al., 2020). Mechanistically, TMAO disrupts myocardial calcium homeostasis through two distinct pathways: direct elevation of intracellular Ca^2+^ concentration and promotion of SERCA2a (sarco/endoplasmic reticulum Ca^2+^-ATPase) autophagic degradation, which attenuates sarcoplasmic reticulum Ca^2+^ reuptake capacity (Lei et al., 2025). This “calcium overload-SERCA2a inactivation-calcium handling disorder” positive feedback loop ultimately drives cardiomyocyte contractile dysfunction and pathological hypertrophy.

In summary, TMAO demonstrates a biphasic protective-damaging mechanism in HF, with its net effect determined by dose thresholds, exposure duration, and pathological context. At the damaging level, high-dose/chronic exposure drives HF progression through synergistic multi-pathway mechanisms: inflammation, oxidative stress, fibrosis, metabolic derangement, and direct cardiac/electrophysiological impairments. Conversely, at the protective level, low-dose/acute exposure mitigates inflammation via TNF signaling inhibition, activates the Nrf2 pathway to induce antioxidant enzymes (e.g., HO-1, NQO1) for oxidative stress resistance, and induces metabolic reprogramming (e.g., shifting from fatty acid oxidation to glucose oxidation under hypoxia) to maintain energy homeostasis. Under specific conditions (e.g., low-concentration exposure or acute stress), TMAO further activates endoplasmic reticulum stress-protective pathways or upregulates antioxidant enzymes, providing compensatory myocardial protection.

#### 3.1.2 PAA and its secondary derivatives

HF promotes gut dysbiosis, characterized by opportunistic pathogen overgrowth, which triggers reprogramming of the phenylalanine metabolic pathway. This reprogramming significantly enriches gene pathways encoding key enzymes for PAA synthesis, which directly enhances the conversion efficiency of phenylalanine (Phe) to PAA. In the human liver, PAA conjugates with glutamine to form PAGln, the primary circulating metabolite in humans. This section investigates the impact of PAGln on HF progression while exploring the potential roles of other PAA-derived metabolites in this pathological context.

##### 3.1.2.1 Multimechanistic pathways of PAA

Aberrant PAA metabolism in HF patients directly drives the pathological progression of HF through multiple mechanisms, including metabolite imbalance, cytotoxic effects, and significantly elevated short-term mortality risk (Chen et al., 2024). First, PAA activates the NOX4 enzyme to enhance mitochondrial H₂O₂ production, triggering oxidative stress responses that result in elevated DNA damage markers, activated DNA damage responses, mitochondrial dysfunction, and endothelial cell senescence—characterized by cell cycle arrest and enhanced aging phenotypes. Second, PAA-induced EC senescence activates the senescence-associated secretory phenotype, which secretes pro-inflammatory cytokines and adhesion molecules that drive persistent inflammation, myocardial fibrosis, and tissue damage. Third, PAA alleviates epigenetic repression of pro-inflammatory genes through CaMKII-dependent HDAC4 phosphorylation and cytoplasmic translocation, leading to dysregulated gene expression and exacerbated cardiomyocyte apoptosis/fibrosis. Fourth, PAA inhibits mitochondrial respiration and glycolytic pathways, reducing ATP production and causing cellular energy deficiency that impairs cardiac pumping capacity (Saeedi Saravi et al., 2025). Yang et al. further elucidate the pathogenic mechanisms: PAA directly induces senescent ECs to secrete SASP components, thereby amplifying chronic vascular inflammation (Yang et al., 2025). Collectively, these interconnected mechanisms form a complex network that drives HF progression through PAA-mediated metabolic and cellular dysfunction.

##### 3.1.2.2 Complex multiple mechanisms of PAGln

PAGln, the primary gut microbiota-derived metabolite of PAA, has been well-characterized in recent studies for its pathological role in HF and associated multidimensional mechanisms. In hypertrophic cardiomyopathy cohorts, plasma PAGln levels decrease significantly after myocardial resection, supporting its potential as a dynamic biomarker for metabolic function improvement (Wolter et al., 2021). In stable HF patients, PAGln levels independently correlate with all-cause mortality. Through β2AR-mediated negative inotropic effects, PAGln inhibits sympathetically induced myocardial contractility enhancement while directly upregulating BNP gene expression in cardiomyocytes, thereby creating a vicious cycle of increased cardiac workload (Tang et al., 2024). PAGln levels show strong positive correlations with BNP (*r* = 0.924, *p* < 0.001), a negative correlation with LVEF (*r* = −0.587, *p* < 0.001), and a positive correlation with the LVEDD/LVESD ratio (*r* = 0.477–0.529, *p* < 0.001) (Zhang et al., 2023), thus serving as an independent predictor of progressive cardiac structural and functional deterioration.

The pathological effects of PAGln exhibit tissue specificity and signaling pathway cross-talk, primarily mediated through β2AR-dependent mechanisms. In cardiomyocytes, PAGln directly induces sarcomere dysfunction via GPCR signaling, attenuating contractility in the presence of sympathetic agonists (Romano et al., 2023). Concurrently, it enhances sympathetic tone through β2AR activation, leading to cardiomyocyte dysfunction, negative inotropy, and exacerbated HF progression (Zhang et al., 2023). Oxidative stress serves as a key pathogenic node, PAGln induces oxidative stress in atrial myocytes (HL-1) via β2AR activation, characterized by elevated ROS production, increased NOX activity, and reduced SOD activity (Fang et al., 2022). This is accompanied by upregulated p-CaMKII, p-RyR2, and RyR2 expression, promoting calcium overload and apoptosis. Through the ADR-AMPK axis-mediated phosphorylation of DRP1 at Ser616, PAGln drives mitochondrial fragmentation, membrane potential depolarization, and ROS accumulation, culminating in DNA damage, cell cycle arrest, and dose-dependent senescence in human cells (Yang et al., 2025). In cardiovascular endothelial injury models, PAGln impairs endothelial proliferation, migration, and angiogenesis through β2AR-LDHA axis inhibition, compromising cardiac function (Zhang et al., 2024). Via the β2AR-cAMP-PKA/NF-κB pathway, PAGln promotes inflammatory cytokine transcription, as evidenced by increased NF-κB phosphorylation and pro-inflammatory macrophage polarization in murine BMDMs following 24-h treatment with 100 μM PAGln (Huang and Zhang, 2023).

In humans, PAGln enhances platelet reactivity and thrombotic potential through ADR signaling, accelerating thrombus formation in arterial injury models while promoting systemic inflammation and endothelial dysfunction to drive atherosclerosis (Fang et al., 2022). Notably, PAGln’s effects are disease-context dependent: in diabetic states, it activates the cAMP/PKA/NF-κB pathway via β2AR to upregulate IL-1β/TNF-*α* and promote M1 macrophage polarization, accelerating atherosclerosis. In non-diabetic models, it induces endothelial injury via NOX2-dependent oxidative stress, increasing NF-κB levels and vascular inflammation/early atherosclerosis (Huang, 2023). Consistent with Allemann et al.’s findings, 72-h PAGln treatment (100 μM) in HAECs significantly elevates ROS levels and activates oxidative stress, with Western blot confirming NOX2 and NF-κB upregulation without affecting NOX4, thus validating the NOX2-NF-κB axis-mediated endothelial oxidative stress injury independent of NOX4 (Allemann et al., 2023).

The biological effects of PAGln adversely impact myocardial electrical activity, cardiac function, and structural remodeling. Mechanistically, PAGln upregulates key proteins in the TLR4/AKT/mTOR pathway while modulating calcium homeostasis through NCX1 upregulation and CACNA1C downregulation. Concurrently, it induces ROS accumulation and oxidative stress, activating the CaMKII signaling pathway. These alterations collectively induce ventricular electrophysiological instability, characterized by a shortened effective refractory period, prolonged sinoatrial node recovery time, and disrupted myocardial electrical activity and rhythm (Fu et al., 2023). In isolated cardiomyocytes, PAGln induces hypercontractility (increased sarcomere shortening fraction) independent of classical AR-PKA signaling. This is accompanied by altered calcium cycling, evidenced by an increased peak calcium transient and elevated diastolic calcium levels (Li et al., 2024). At the structural level, PAGln significantly increases left atrial diameter, reduces left ventricular ejection fraction, exacerbates atrial fibrosis (increased Masson staining fibrosis ratio), and induces cardiomyocyte hypertrophy (Fu et al., 2024). Moreover, PAGln aggravates myocardial ischemia-induced intestinal barrier dysfunction and promotes atrial fibrosis/electrophysiological instability. Notably, PAGln exacerbates atrial remodeling through ferroptosis pathway activation (with concomitant ROS accumulation) and NLRP3 inflammasome activation (Wang et al., 2025).

Paradoxically, emerging evidence suggests that PAGln may exhibit beneficial effects under specific conditions. For instance, PAGln significantly alleviates doxorubicin-induced cardiac toxicity via gut microbial metabolism regulation, reverses abnormal myocardial gene expression (e.g., Klb, Ece2, Casq1), and mitigates myocardial injury and apoptosis through mechanisms involving lipid metabolism, calcium signaling pathways, and store-operated Ca^2+^ channel activity (Huang et al., 2025).

Importantly, early studies often conflated phenylacetylglycine (PAGly) with PAGln due to structural similarity. However, modern high-resolution metabolomics has definitively distinguished their metabolic pathways and pathological associations. While PAGly research shows promise in preclinical models, its translational potential across species requires further validation. Future studies must rigorously differentiate between PAGln and PAGly while further investigating PAGly’s mechanisms in human cardiovascular diseases.

##### 3.1.2.3 Dual action of benzoic acid

The biotransformation of benzoic acid and its derivatives is closely associated with cardiovascular health. Anaerobic bacteria metabolize PAA into benzoic acid via specific metabolic pathways. While clinical studies directly linking this process to HF remain limited, preclinical research has identified diverse mechanisms of action. Benzoic acid exhibits toxicity through mitochondrial thiol binding, inhibition of mitochondrial respiratory chain complex I, and excessive ROS production, thereby exacerbating oxidative stress and disrupting energy metabolism—key contributors to HF pathogenesis (Beloborodova et al., 2012).

In contrast, certain derivatives demonstrate protective effects. For instance, 2-(3-(Chloromethyl)benzoyloxy)benzoic acid shows significant anti-inflammatory and antioxidant properties in LPS-induced mouse models. it reduces prostaglandin E2 synthesis via COX-2 inhibition, downregulates the NOX2-NF-κB pathway, and suppresses ROS generation, outperforming aspirin (Tjahjono et al., 2024). R-/S-2-(2-Hydroxypropylamido)benzoic acid exerts antiplatelet effects by modulating the thromboxane A2 (TXA2)/prostacyclin balance. This compound inhibits COX-1 activity, reduces TXA2 production, and counteracts ADP/collagen-induced platelet aggregation. Its antithrombotic efficacy is comparable to aspirin without inducing cellular toxicity (Zhang et al., 2017).

In summary, the effects of PAA and PAGln on HF exhibit dualistic characteristics, with outcomes determined by dose, exposure duration, and pathological context. Mechanistically, PAA induces oxidative stress, promoting endothelial senescence and pro-inflammatory cytokine release, thereby driving fibrosis and metabolic collapse. PAGln inhibits myocardial contraction via β2AR signaling, while concurrently activating pathways that exacerbate oxidative stress, calcium dysregulation, and electrophysiological abnormalities, ultimately accelerating thrombosis and structural remodeling. Notably, at low doses or under specific pathological conditions (e.g., doxorubicin-induced injury), PAGln demonstrates protective effects through gut microbial metabolism regulation, reversing abnormal gene expression and attenuating myocardial injury. Benzoic acid derivatives exhibit anti-inflammatory, antioxidant, and antiplatelet properties. Condition specificity is manifested through dose-dependent effects (protective at low doses, damaging at high doses) and pathological differences.

#### 3.1.3 LPS

The gut microbiota-derived metabolite LPS translocates into systemic circulation in HF patients via intestinal barrier dysfunction, triggering systemic inflammation and exacerbating cardiac injury. Serum LPS levels are inversely correlated with cardiac function parameters (Sandek et al., 2012). HF-induced gut dysbiosis promotes LPS overproduction. Current studies demonstrate that LPS exerts multidimensional pathological effects on the heart, including calcium dysregulation, neurohumoral imbalance, oxidative stress, autophagy impairment, and cross-activation of signaling pathways. Collectively, these findings elucidate the molecular mechanisms underlying LPS-mediated HF pathogenesis, providing critical insights into the role of gut microbiota–host interactions in cardiovascular disease and establishing a foundation for targeted therapeutic strategies.

##### 3.1.3.1 Cardiac function and cardiac nerve damage

LPS, a key component of Gram-negative bacterial cell walls, induces cardiac dysfunction by disrupting myocardial electrical activity and neural regulation, with the magnitude of its effects modulated by baseline disease status, sex differences, and individual adaptive responses. Early studies using isolated rat heart models demonstrated that LPS inhibits the sarcoplasmic reticulum Ca^2+^ uptake rate and reduces the P-PLB/total PLB ratio, while increasing serine/threonine protein phosphatase activity, promoting PLB dephosphorylation. This disrupts normal calcium cycling in cardiomyocytes, particularly during diastole, resulting in impaired Ca^2+^ transport and excitation-contraction coupling, as evidenced by reduced contraction amplitude, left ventricular developing pressure, and an impaired force-frequency relationship (Joulin et al., 2009). These findings were corroborated by Wagner et al., who identified LPS-specific impairment of diastolic Ca^2+^ clearance mechanisms. LPS induces dual damage to systolic and diastolic function by inhibiting Na^+^/Ca^2+^ exchanger and plasma membrane Ca^2+^-ATPase activity, causing intracellular Ca^2+^ overload, and by reducing myofilament Ca^2+^ sensitivity and actomyosin sliding efficiency (Wagner et al., 2015).

At the neural level, LPS activates paraventricular nucleus microglia, reducing GABAergic inhibition of PVN-RVLM neurons (as indicated by decreased sIPSC frequency) and disinhibiting GABAA receptor-mediated suppression, thereby enhancing sympathetic hyperactivity (Han et al., 2021). Concurrently, LPS induces TNF-*α* release and potentiates splanchnic/splenic sympathetic outflow, accelerating heart rate (Martelli et al., 2014). Immune-vagal interactions are evident in LPS cardiotoxicity. In an LPS-induced acute lung injury model, dynamic vagal modulation of cardiac function was observed, with significant increases in HRV indices (SDRR, RMSSD, HF) at 120–180 min, indicating complex immune-vagal crosstalk (Zila et al., 2023). At the molecular level, LPS binds TLR4 to activate the canonical NF-κB pathway, upregulating myocardial NOS2 expression and increasing cGMP levels, thereby suppressing contractility (Nemoto et al., 2002). LPS also activates TLR4 to induce intrinsic cardiac adrenergic cells to secrete norepinephrine, which activates CaMKII via β1-AR, triggering NF-κB/MAPK pathways and forming a positive feedback loop of TNF-*α* secretion and cardiac dysfunction (Yang et al., 2022). Additionally, LPS upregulates MKP-1 via protein kinase C, inhibiting Ang II-induced MAPK activity and modulating cardiac fibroblast function (Stawowy et al., 2003).

##### 3.1.3.2 Oxidative stress and autophagy imbalance

LPS orchestrates a complex pathological network by inducing oxidative stress and autophagy imbalance. The temporal kinetics of this process were first elucidated by Tamion et al., who demonstrated that LPS induces early-phase cardiac dysfunction via oxidative stress (Tamion et al., 2010)—a concept that has been validated across multiple experimental models. Central to this pathophysiology is TLR4-dependent signaling. LPS binding to TLR4 triggers intracellular signaling cascades, including the release of NO and TNF-*α*, activation of ERK/STAT pathways, and engagement of the canonical NF-κB/MAPK pathways. As the primary pattern recognition receptor for pathogen-associated molecular patterns, TLR4’s canonical ligand, LPS, induces cytokine secretion, which is significantly attenuated by pharmacological TLR4 blockade (Panaro et al., 2010).

Oxidative stress is manifested through NOX2/ROS signaling, leading to excessive ROS production, lipid peroxidation, SOD inhibition, and a redox imbalance between glutathione and oxidized glutathione (Skibska et al., 2022). These biochemical alterations synergistically contribute to the exacerbation of cardiac dysfunction. A bidirectional regulation between oxidative stress and autophagy is exemplified by LPS-induced recruitment of PINK1/Parkin (PARK2) to damaged mitochondria, facilitating mitophagy via mitochondrial outer membrane protein ubiquitination. When mitophagy is impaired (e.g., in PARK2 deficiency), compensatory macroautophagy is enhanced. Concurrent mitochondrial dysfunction includes reduced respiratory chain activity, disrupted oxidative phosphorylation, and increased H₂O₂ release, though these effects are partially mitigated by PARK2-mediated preservation of mitochondrial Ca^2+^ retention capacity and NF-κB-driven OPA1 expression (Piquereau et al., 2013).

Protein quality control is also targeted by TLR4 signaling, which inhibits 20S/26S proteasome activity while modulating Akt/FoxO3a and p38 MAPK pathways. This leads to the upregulation of muscle RING-finger protein 1 and muscle atrophy F-box expression, activating autophagy and culminating in myocardial protein degradation (Jamart et al., 2014). Autophagy exhibits dual roles in LPS cardiotoxicity: cytoprotective autophagy via the TNF-*α*/p38MAPK/NOS axis, where oxidative stress triggers LC3-II conversion to limit LPS-mediated ROS overproduction (Yuan et al., 2009). and ferroptotic injury mediated by nuclear receptor coactivator 4 (NCOA4)-dependent ferritin degradation, where LPS upregulates NCOA4, promoting ferritin sequestration into autophagosomes via sideroflexin 1-dependent iron transport. This exacerbates mitochondrial ROS generation and lipid peroxidation (Li et al., 2020). Additionally, apoptotic and necroptotic components further complicate the pathology, as LPS suppresses the anti-apoptotic protein Bcl-2 while upregulating caspases-3/9 (Dong et al., 2013).

##### 3.1.3.3 Myocardial inflammatory response

In cardiomyocytes, LPS induces inflammatory cascades via dual pathways: the Ca^2+^-sensing receptor-phospholipase C-inositol trisphosphate axis promotes Ca^2+^ influx, activating Ca^2+^/CaMK/PKC signaling to release TNF-*α* and IL-6, establishing an inflammatory-calcium overload positive feedback loop (Wang et al., 2013). Concurrently, TLR4 activation engages the MyD88/TRIF-NF-κB/MAPK axes to upregulate inflammatory cytokines and iNOS expression. The excessive production of NO exacerbates cardiomyocyte toxicity, promoting apoptosis, hypertrophy, and fibrosis (Du et al., 2017). Additionally, LPS activates the STING-IRF3 pathway, inducing NLRP3 inflammasome assembly and ROS-TXNIP-dependent oxidative stress, which triggers caspase-1-mediated pyroptosis and releases IL-1β/IL-18 (Li et al., 2019). In atrial tissue, LPS promotes NET release through the activation of inflammatory-immune axes, leading to local microenvironment disruption and contributing to atrial remodeling (Xiang et al., 2024). Dose-dependent effects are evident in this context: low-dose LPS (1 ng/mL) induces the secretion of lipopolysaccharide-binding protein, serum amyloid A, and RANTES through adipocyte-macrophage interactions. This response is mediated by the TNF-α/p38MAPK/NOS axis (Nakarai et al., 2012). In pathological co-models, LPS pro-inflammatory effects are amplified. For example, under conditions of high glucose combined with hypoxia/reoxygenation, LPS activates caspase-1-dependent pyroptosis via the ROS-NLRP3 axis, accompanied by lactate dehydrogenase leakage and inflammatory cytokine bursts (Qiu et al., 2019). It also upregulates cytochrome P450 1B1 expression via TLR4-NF-κB/MAPK activation. Toxic metabolites, such as hydroxyeicosatetraenoic acid from arachidonic acid metabolism, further exacerbate cardiac hypertrophy and failure (ElKhatib et al., 2024).

NLRX1 ubiquitination is induced by LPS, and NLRX1 deficiency exacerbates cardiac injury by enhancing NF-κB and NLRP3 inflammasome activation, while diminishing the protective effects of CYP2J2/epoxyeicosatrienoic acid. This leads to elevated pro-inflammatory cytokines, mitochondrial dysfunction, and ROS accumulation (Zhao et al., 2020). Furthermore, LPS upregulates macrophage Fcα/*μ* receptor expression through p38MAPK/NF-κB signaling, promoting the internalization of natural anti-oxLDL IgM immune complexes and accelerating foam cell formation, a key process in atherosclerosis (Feng, 2011). LPS also activates mTOR, phosphorylating its substrate rpS6 to promote IκB-α degradation, releasing NF-κB p65 into the nucleus. This triggers the expression of iNOS, COX-2, and TNF-α, leading to excessive NO and prostaglandin production, and contributing to myocardial inflammation (Temiz-Resitoglu et al., 2017).

Notably, LPS cardiotoxicity exhibits disease specificity. In a cirrhotic rat model, LPS does not induce tachycardia in rats but causes biphasic changes in heart rate variability, with preserved atrial cholinergic responses, indicating the development of tolerance in cirrhotic conditions (Haddadian et al., 2013). Recent studies also reveal sex differences in mitochondrial responses: post-LPS exposure, male mice show upregulation of mitochondrial complexes I, III, and V proteins, while females exhibit downregulation of complexes III and IV. This sex-specific regulation of oxidative phosphorylation proteins renders male hearts more sensitive to LPS, manifesting as rapid functional suppression, while female hearts display milder and delayed suppression (Harvey et al., 2025). Dose-dependent effects are also observed: low-dose LPS enhances integrin αMβ2 complex function, promoting leukocyte migration and adhesion, which optimizes inflammatory signaling and reduces myocardial I/R injury, thereby exerting cardioprotective effects (Wu et al., 2019; Lichte et al., 2013).

In summary, LPS induces multi-dimensional cardiac toxicity. Notably, LPS displays dose-dependent biphasic effects: at pathogenic levels, it drives HF progression through TLR4-mediated signaling cascades and mitochondrial dysfunction. However, at subpathogenic doses, LPS may exert compensatory effects. Context-specific modulation is observed in sex differences in mitochondrial response: male hearts exhibit rapid functional suppression, while female hearts show more delayed suppression. These findings emphasize the need for precision strategies that consider dose thresholds, exposure duration, and patient-specific pathology to effectively modulate LPS’s net pathological effect in HF pathogenesis.

#### 3.1.4 Other cardiotoxic metabolites

Beyond the three classical metabolites discussed, other derivatives significantly contribute to HF progression. Microbiota-derived imidazole propionic acid (IMP), a histidine metabolite, shows significantly elevated serum levels in HF patients, correlating with left ventricular dysfunction, increased NT-proBNP, and higher 5-year all-cause mortality, suggesting its potential as an independent predictive biomarker (Molinaro et al., 2023). Mechanistically, IMP activates the mTORC1 pathway while inhibiting AMPK signaling, leading to myocardial energy metabolism reprogramming and accelerated fibrosis. IMP is also associated with elevated systemic inflammation markers (CRP) and intestinal barrier dysfunction indicators (intestinal fatty acid-binding protein), potentially exacerbating cardiac inflammation via the gut-heart axis (Raju et al., 2024). Notably, IMP’s inhibitory effect on angiogenesis further impairs myocardial function, creating a vicious cycle with increased intestinal permeability (Dahl et al., 2025). Cholesterol metabolites exhibit dual regulatory effects. Oscillibacter species generate oxysterols via conserved cholesterol-metabolizing enzymes, whose increased abundance correlates with reduced LDL-C/TG levels and elevated HDL-C levels, suggesting potential lipid metabolism regulation to improve cardiac conditions. However, the cardiovascular effects of these metabolites require evaluation in the context of host metabolic status, as some oxysterols may exert protective effects through anti-inflammatory mechanisms, while overall dysbiosis of gut microbial metabolic activity may drive harmful metabolite accumulation (Li et al., 2024). N, N, N-Trimethyl-5-Aminovaleric Acid (TMAVA) impairs cardiac function by inhibiting *γ*-butyrobetaine hydroxylase (BBH, EC 1.14.11.1), disrupting carnitine synthesis, and blocking the organic cation/carnitine transporter 2. This reduces myocardial carnitine uptake and cardiac acylcarnitine levels, ultimately inhibiting fatty acid oxidation (Zhao et al., 2022). The resulting metabolic disturbances cause myocardial lipid accumulation, mitochondrial dysfunction, and ROS accumulation, manifesting as cardiac hypertrophy, reduced contractile function, and impaired exercise tolerance, establishing TMAVA as an independent cardiovascular toxic molecule. Indoxyl sulfate induces oxidative stress and inflammation by activating ERK/p38 MAPK, NF-κB pathways, and the RAS. It also inhibits NOS activity and downregulates cardioprotective factors erythropoietin and Klotho, leading to myocardial contractile dysfunction and rhythm disturbances (Imazu et al., 2017). Collectively, these findings delineate a complex network through which gut microbiota metabolites influence cardiac health via multiple pathways—including energy metabolism disruption, inflammatory activation, and signaling pathway modulation—providing novel therapeutic targets for HF intervention.

### 3.2 The deficiency of protective metabolites and myocardial repair dysfunction

#### 3.2.1 SCFAs

SCFAs are organic fatty acids with carbon chain lengths of 2–6, with acetate, propionate, and butyrate being the primary components, collectively accounting for more than 90% of intestinal SCFAs. Patients with HF often exhibit reduced circulating levels of SCFAs. While most SCFAs exert cardioprotective effects against HF, some studies report potential adverse impacts.

##### 3.2.1.1 Acetate

Acetate exerts cardiac regulation through direct myocardial effects, modulation of energy metabolism, antioxidant stress, and systemic protection via the gut microbiota-host axis. It directly influences cardiac function through dual mechanisms: reducing sympathetic tone to decrease heart rate, and acting on cardiomyocytes via GPCRs to induce negative inotropic effects, manifesting as reduced contractility parameters (Poll et al., 2021).

In energy metabolism, acetate specifically inhibits acetyl-CoA synthetase activity in myocardial fatty acid metabolism pathways, reducing mitochondrial ATP production and directly decreasing myocardial ATP concentration and contractility (Jacob et al., 1997). This mechanism operates independently of calcium regulation, primarily by disrupting fatty acid oxidation-related energy metabolism. At the molecular signaling level, acetate activates GPR41 and GPR43 to inhibit TGF-*β*1-induced SMAD2 phosphorylation, blocking the TGF-β1/SMAD2 pathway (Lin et al., 2022). This reduces cardiac fibroblast-to-myofibroblast transformation and collagen synthesis, ultimately preventing cardiac fibrosis. Additionally, acetate neutralizes acidic environments and inhibits aluminosilicate formation, blocking pathogenic calcium salt generation and alleviating acid-induced cellular damage, thereby reducing cardiovascular pathological risk (Shi et al., 2019).

Indirectly, acetate protects the heart through gut microbiota and metabolic pathways. It remodels the gut microbiota by increasing the abundance of acetate-producing bacteria and decreasing the Firmicutes/Bacteroidetes ratio, modulating cardiac gene expression—upregulating antifibrotic genes and downregulating profibrotic factors. It also improves cardiovascular function by regulating circadian rhythms and the RAAS (Marques et al., 2017). Furthermore, acetate restores taurine-conjugated bile acid balance, inhibits the intestinal-hepatic farnesoid X receptor (FXR)-fibroblast growth factor 15 pathway, and reduces obesity-related metabolic disorders induced by high-fat diets. By controlling weight gain and improving lipid profiles, acetate lowers obesity-induced cardiac injury risk (Wang et al., 2022).

##### 3.2.1.2 Propionate

Propionate optimizes metabolic flux to enhance myocardial function while potentially inducing pathological damage through metabolic abnormalities, thus achieving bidirectional regulation of myocardial injury in HF. At the metabolic level, propionate generates propionyl-CoA and methylmalonyl-CoA, triggering mitochondrial CoA sequestration to directly inhibit fatty acid oxidation and promote metabolic switching to glucose utilization (Wang et al., 2018). This reprogramming may reduce HF risk by enhancing energy utilization efficiency. However, propionate and its metabolites suppress the tricarboxylic acid cycle, reducing ATP production while inducing oxidative stress and inflammatory responses, ultimately impairing mitochondrial function, membrane stability, and contractile function (Riemersma et al., 2017).

Beyond metabolic interventions, propionate activates GPR41 to inhibit the Ang II-caveolin-1 interaction during reperfusion, upregulating angiotensin-converting enzyme 2 expression to mitigate myocardial I/R injury (Deng et al., 2022). It also upregulates cardiac-enriched microRNAs, creating a vicious cycle involving abnormal myosin heavy chain expression, autophagy inhibition, and impaired mitochondrial fatty acid oxidation (Álvarez et al., 2023). Propionate-induced histone propionylation/acetylation downregulates the cyclic guanosine monophosphate-protein kinase G pathway, exacerbating diastolic dysfunction (Park et al., 2023). Direct ion channel effects are confirmed: propionate restores mitochondrial membrane potential in Akt2-deficient cardiomyocytes via GPR41-dependent pathways, improving contractile function (Li et al., 2012). Conversely, it prolongs cardiomyocyte action potential duration, inducing long QT syndrome (Rodriguez-Gonzalez et al., 2018). through mitochondrial ROS-mediated SERCA2a oxidation, it reduces ATPase activity, causing reduced systolic Ca^2+^ release and diastolic Ca^2+^ leakage, triggering arrhythmias (Tamayo et al., 2020).

Notably, chronic propionate accumulation downregulates cGMP-PKG, reducing contractility and promoting diastolic dysfunction with remodeling risk, particularly in female subjects (Park et al., 2023). This sex difference may be related to the *in vivo* regulation of estrogen and progesterone, or the interaction between hormones and propionate.

##### 3.2.1.3 Butyrate

Butyrate enhances cardiac function via non-*β*-adrenergic receptor pathways while simultaneously inhibiting calcium overload and oxidative stress during I/R injury through modulation of SERCA2a activity and mitochondrial function (Seefeldt et al., 2024). Its anti-inflammatory and metabolic remodeling effects are characterized by inhibition of the NF-κB/STAT pathway, suppression of M1 macrophage polarization, and promotion of IL-10-producing M2 macrophage polarization to reduce inflammatory cytokine release. Additionally, it enhances mitochondrial biogenesis via the PGC-1α/TFAM pathway, thereby restoring mitochondrial membrane potential and ATP synthesis, and ultimately inhibits myocardial fibroblast activation and collagen deposition by downregulating the TGF-β/Smad and CTGF pathways (Li et al., 2022). For immunoregulation, butyrate activates GPR41/43/109A receptors to promote Treg differentiation and, as a histone deacetylase inhibitor, remodels chromatin structure, upregulates anti-fibrotic gene expression, and directly inhibits TGF-β signaling to reduce COL1A1 expression at both mRNA and protein levels (Wang et al., 2022).

Structural optimization has improved butyrate’s stability and clinical application prospects. Its derivative, phenylbutyric acid, exhibits similar protective effects, including maintenance of antioxidant enzyme activity via inhibition of mitochondrial ROS accumulation and H₂O₂ release, reduction of myocardial apoptosis through downregulation of iNOS/nitrotyrosine expression, and attenuation of collagen deposition by inhibiting the CTGF/MMP-2 pathway (Russo et al., 2019). Sodium butyrate acts as an HDAC inhibitor to increase histone acetylation levels, thereby downregulating cardiac hypertrophy markers (β-MHC) and proto-oncogenes (c-fos/c-jun), while modulating MMP-2/9 and TIMP-1/2 expression to alleviate inflammation (Subramanian et al., 2016). These mechanisms synergistically establish a comprehensive protective network spanning gene expression regulation to cellular functional repair.

In summary, patients with HF have reduced circulating levels of SCFAs, which directly compromise their cardiovascular protective effects. The characteristics of SCFAs are defined by component-specific bidirectional regulation and multidimensional synergism: acetate modulates cardiac function through direct myocardial effects (e.g., negative inotropy) and metabolic regulation. Propionate exhibits metabolic duality—enhancing the metabolic shift toward glucose utilization for improved energy efficiency while potentially inducing oxidative stress and arrhythmias (e.g., prolonged QT interval) via inhibition of the tricarboxylic acid cycle. Butyrate strengthens cardiac function via non-β-adrenergic pathways and demonstrates integrated protective properties, including anti-inflammatory, anti-fibrotic, and epigenetic modulation (e.g., histone deacetylase inhibition). Overall, SCFAs play critical regulatory roles in HF pathogenesis through coordinated metabolic-inflammatory-epigenetic pathways, with component-specific bidirectional modulation as the core mechanism, positioning them as promising therapeutic targets for cardiovascular diseases.

#### 3.2.2 BA and secondary BAs

As pivotal messengers bridging the gut microbiota and the host cardiovascular system, the BA family—including primary and secondary BAs—forms a multifaceted regulatory network through activation of nuclear receptors such as TGR5, FXR, and PXR. HF leads to reduced BA secretion and diminished conversion to secondary BAs, with mechanisms showing marked heterogeneity: certain BAs exert cardioprotective effects through anti-inflammatory and antioxidant pathways, while others may induce cardiac injury at specific dose levels. We summarized their dual roles and explored their clinical translation potential.

##### 3.2.2.1 Multiple effects of BA

BAs remodel myocardial energy substrate utilization through epigenetic modifications and regulation of key metabolic enzymes. In Fxr/Shp double-knockout mice, BA accumulation inhibits Pgc1α transcriptional activity and downregulates core fatty acid oxidation genes (e.g., Cpt1b, Acadl), thereby reducing mitochondrial β-oxidation rates. Concurrently, it reverses Pdk4-mediated suppression of glucose oxidation, triggering adaptive substrate switching from fatty acids to glucose (Desai et al., 2017). BA activates the TGR5-mediated AKT/GSK3β pathway, inhibiting GSK3β activity to promote cardiomyocyte growth gene expression and induce pathological hypertrophy, while suppressing FAO and upregulating glycogen synthesis (Desai et al., 2010). Guan et al. revealed that BA protects cardiomyocytes from hyperglycemia-induced injury via TGR5-dependent inhibition of the NF-κB pathway and pro-inflammatory cytokines. Concurrently, FXR activation represses endothelin-1 transcription and AngII signaling to attenuate metabolic cardiac damage (Guan et al., 2022). Furthermore, BA-gut microbiota interactions generate secondary BAs through BSH enzyme metabolism. These metabolites suppress NLRP3 inflammasome activation via TGR5, thereby reducing systemic inflammation. Notably, BA exhibits dual cardiotoxic mechanisms: direct disruption of cardiomyocyte calcium dynamics, causing electrophysiological instability, or promotion of fibroblast-to-myofibroblast transition, which reduces cardiomyocyte membrane potential and slows conduction through gap junctions (Miragoli et al., 2011).

##### 3.2.2.2 Potential applications of secondary BAs

Hydroxydeoxycholic acid (HDCA) exerts cardioprotective effects by regulating cardiac inflammation and structural remodeling. Through TGR5 receptor activation, it inhibits STAT3/NF-κB/MAPK pathway activation, reduces pro-inflammatory cytokine release, and improves cardiac function parameters at 100 mg/kg doses (Wang et al., 2025). Synthetic derivatives, such as INT-777, enhance survival kinases (Akt/ERK/PKA) and heat shock proteins via TGR5, optimizing glucose metabolism (Vítek, 2017).

Chenodeoxycholic acid (CDCA) primarily modulates atrial structural remodeling in HF. In atrial fibrillation patients, elevated circulating CDCA correlates with left atrial low-voltage zone size. *In vitro*, CDCA induces mouse atrial myocyte apoptosis in a dose-dependent manner (Wang et al., 2020). Both CDCA and deoxycholic acid (DCA) demonstrate positive inotropic effects (enhanced LVDP, dp/dt max, muscle strip tension) and negative chronotropic effects in isolated rat hearts via calcium-dependent mechanisms (Gao et al., 2021).

Cholic acid (CA) activates TGR5, inducing phosphorylation of Akt/ERK1/2/PKA while upregulating heat shock proteins (HSP32/27/72/90) and eNOS, forming a cardioprotective network. CA and INT-777 inhibit FOXO-1 transcriptional activity, downregulating PDK4 to enhance glucose metabolic flexibility (Eblimit et al., 2018).

Deoxycholic acid (DCA) enhances myocardial contractility via increased left ventricular posterior wall thickness, septal thickness during systole, and stroke volume, thereby elevating cardiac output and mean arterial pressure without altering systemic vascular resistance (Nowiński et al., 2023). In autonomic regulation, DCA initially reduces heart rate via reflex mechanisms but subsequently activates sympathetic pathways to maintain HR. Via TGR5, DCA upregulates PKA signaling, inhibits NF-κB p65 phosphorylation, and reduces cytokine release and ROS generation, exerting anti-inflammatory and antioxidant effects under hypoxia (Huang et al., 2023). Notably, DCA exhibits dose-dependent effects: 50–100 μM may transiently impair contractility, requiring strict dosage control.

Lithocholic acid (LCA) inhibits high glucose-induced cardiomyocyte hypertrophy via TGR5-PKA signaling, offering therapeutic potential for diabetic cardiomyopathy (Cheng et al., 2019). It modulates cardiac protection via regulation of EphA2 tyrosine phosphorylation. In doxazosin-induced HL-1 apoptosis models, LCA suppresses EphA2 phosphorylation in a concentration-dependent manner, blocking SHP-2-mediated pro-apoptotic signaling while upregulating total EphA2 expression (Jehle et al., 2012).

Ursodeoxycholic acid (UDCA) exerts multi-level cardioprotection. Clinically, it improves peripheral hemodynamics and hepatic function in CHF patients, evidenced by increased peak blood flow after limb ischemia and reduced sTNFR-1 levels (von Haehling et al., 2012). Mechanistically, UDCA activates K_ATP channels to hyperpolarize myofibroblast membrane potential, counteracting TC-induced depolarization. It inhibits hypoxia-driven fibroblast-to-myofibroblast transition, reducing abnormal electrical coupling (Schultz et al., 2016). Additional studies confirm that UDCA enhances K^+^ efflux for membrane hyperpolarization, directly suppressing calcium dysregulation and electroconduction inhibition while reducing fibroblast-to-myofibroblast conversion (Miragoli et al., 2011). Via TGR5, UDCA inhibits ERK1/2 phosphorylation, blocking the non-canonical TGF*β*/WWP2 pathway to downregulate fibrosis-related genes (COMP, FOXP2) and reduce collagen I/VI synthesis (Reilly-O'Donnell et al., 2024). At the molecular level, UDCA suppresses mitochondrial ROS production and upregulates antioxidant enzymes, preventing cytochrome c release and apoptotic cascades (Hanafi et al., 2017). It also inhibits neutral sphingomyelinase activity, reducing ceramide generation to block apoptosis while improving myocardial energy metabolism (Hanafi et al., 2016). Furthermore, UDCA inhibits hypoxia-inducible factor hyperactivation, stabilizing calcium dynamics and preventing hypoxia-induced signaling dysfunction (Mohamed et al., 2017).

In summary, the core functional traits of BAs are defined by bifunctional regulation: certain BAs confer cardioprotection through metabolic remodeling (e.g., substrate switching from fatty acids to glucose), anti-inflammatory/antioxidant actions, and structural modulation (e.g., fibrosis inhibition, contractility enhancement). others may induce cardiac injury at specific doses (e.g., disrupting calcium dynamics, promoting fibroblast-to-myofibroblast transition). Secondary BAs, generated through gut microbiota-mediated dehydroxylation, display distinct profiles: HDCA improves cardiac function via inflammation reduction. UDCA exerts multi-level protection (hemodynamic improvement, fibrosis inhibition, oxidative stress reduction). and CDCA may drive atrial remodeling and apoptosis. Overall, BAs and their secondary metabolites function as dynamic regulators in HF pathogenesis, with component-specific protective and detrimental effects and dose-dependent characteristics, underscoring their therapeutic potential and the need for precise dosage control.

#### 3.2.3 H₂s

H₂S is a gaseous signaling molecule crucial for cardiovascular homeostasis. The gut microbiota modulates H₂S production and metabolism by regulating cystathionine-*γ*-lyase (CSE) activity, while HF disrupts the CSE/H₂S signaling axis, impairing H₂S production and reducing its normal function. H₂S mediates cardioprotective effects through dual mechanisms: conventional pathways (e.g., antioxidant response, mitochondrial bioenergetics enhancement) and novel processes, including epigenetic regulation and inhibition of pathological programmed cell death, thereby maintaining cardiovascular homeostasis.

##### 3.2.3.1 Oxidative stress regulation and mitochondrial protection

H₂S mediates cardioprotection through dual antioxidant mechanisms: direct ROS scavenging and enhancement of the endogenous antioxidant system, thereby establishing a synergistic defense network for oxidative stress regulation and mitochondrial protection. Specifically, H₂S reduces superoxide anion generation by inhibiting mitochondrial complex IV activity, while upregulating MnSOD and Cu/Zn-SOD to achieve coordinated control of ROS sources and terminal clearance (Sun et al., 2012).

Under specific pathological conditions, H₂S shows more targeted effects. In doxorubicin -induced cardiomyopathy models, H₂S induces S-sulfhydration of KEAP1, promoting NRF2 nuclear translocation and activating the SLC7A11/GSH/GPx4 axis to reduce lipid peroxidation, with efficacy directly correlated with ferroptosis inhibition (Zhang et al., 2024). H₂S also increases AKT/FOXO3a phosphorylation while reducing Bim expression to inhibit apoptosis (Liu et al., 2016). In adriamycin-induced models, H₂S attenuates mitochondrial swelling and restores myocardial/plasma H₂S levels, improving left ventricular systolic function (Su et al., 2009). In hypertensive hypertrophy models, H₂S regulates the *α*/β-MHC ratio, reduces the HW/BW ratio and cardiomyocyte cross-sectional area, enhances total antioxidant capacity, and decreases MDA levels, exerting antioxidative and anti-hypertrophic effects (Huang et al., 2017). LaPenna et al. summarized three core pathways: NRF2/NRF-1 activation for oxidative stress inhibition, eNOS-NO axis enhancement for vasodilation, and RAAS suppression for afterload reduction (LaPenna et al., 2021), collectively forming an integrated protective network.

##### 3.2.3.2 Improved myocardial metabolism

H₂S modulates myocardial metabolism through three key mechanisms: lipid metabolism reprogramming, mitochondrial function preservation, and hypoxia adaptation regulation. In diabetic cardiomyopathy models, H₂S activates the APN-AMPK signaling axis, upregulating p-AMPK, GLUT4, and p-ACC expression to enhance fatty acid oxidation and glucose uptake while reducing lipid accumulation. It also inhibits ATF6 cleavage, IRE1/XBP1 splicing, and JNK phosphorylation, thereby mitigating ER stress-induced damage and creating metabolic-stress synergy (Barr et al., 2015). In HFpEF models induced by a high-fat diet plus L-NAME, the H₂S donor NaHS reversed inhibition of the PGC-1α-NRF1-TFAM pathway and restored mitochondrial ultrastructure and oxidative phosphorylation function (Huang et al., 2023). Under hypoxic conditions, H₂S regulates HIF-1α through dual pathways: it reversibly reduces cellular oxygen consumption to accelerate VHL-mediated HIF-1α ubiquitination and degradation, while decreasing HIF-1α stability to inhibit downstream genes (e.g., VEGF, GLUT1) when mitochondrial function is preserved (Kai et al., 2012). This metabolic adaptation enables H₂S to balance oxygen consumption and energy production in hypoxic environments.

##### 3.2.3.3 Anti-fibrosis and autophagy regulation

H₂S mediates antifibrotic effects through multi-pathway synergy. It inhibits the Warburg effect by reducing pyruvate dehydrogenase kinase 4 /LDHA expression, lactate accumulation, and ATP depletion, thereby blocking glycolytic reprogramming. Concurrently, H₂S suppresses atrial collagen deposition and myocyte apoptosis by inhibiting ER stress pathways (Hu et al., 2021). Mechanistically, H₂S induces S-sulfhydration of SIRT3 to inhibit TGF-β1/Smad2/3 signaling, reducing collagen I/III production and fibroblast proliferation/migration (Hu et al., 2024). It also activates PI3K/AKT/eNOS signaling to reduce atrial oxidative stress/inflammation, inhibit cardiac fibroblast activation, and alleviate fibrosis (Xue et al., 2020). In a pressure overload-induced HF model, the H₂S donor diallyl trisulfide improved cardiac function through three mechanisms: (1) promoting angiogenesis and myocardial vascularity, (2) activating the eNOS-NO axis to enhance NO bioavailability, and (3) enhancing antioxidant defense to inhibit left ventricular remodeling and improve systolic function (Polhemus et al., 2013).

Regarding autophagy regulation, H₂S exhibits model-specific dual effects: in I/R injury, it activates AMPK/mTOR to restore autophagic flux (Zhang et al., 2018). in smoking models, it inhibits JNK/p38 MAPK to reduce caspase-3 activation and myocyte apoptosis (Zhou et al., 2014). H₂S also modulates apoptosis via JAK2/STAT3 phosphorylation, upregulating Bcl-2 and downregulating Bax to block mitochondrial apoptosis (Luan et al., 2012). The Na₂S donor reduces myocardial ASC expression to inhibit persistent NLRP3 inflammasome activation and scar formation (Nguyen et al., 2020). For necroptosis, H₂S inhibits RIP1/RIP3/MLKL-mediated pathways to reduce myocardial death and fibrosis (Ma et al., 2022). ER and sarcoplasmic reticulum stress regulation complements these effects: H₂S pretreatment downregulates GRP78/CHOP/ATF6 expression induced by I/R, blocking pro-apoptotic ER stress signaling (Li et al., 2015).

##### 3.2.3.4 Regulates the neuroendocrine system

H₂S mediates cardiac structural homeostasis and functional recovery after HF through an integrated neuroendocrine regulatory network. Key mechanisms include inhibition of the sympathetic-RAAS axis, modulation of neuroreflex pathways, and maintenance of calcium homeostasis. Through dual mechanisms—direct suppression of RAAS overactivation and enhancement of neuroreflex pathways—H₂S reduces renal norepinephrine, plasma renin, and circulating AngII levels, thereby decreasing left ventricular size and preserving ejection fraction (Li et al., 2018). In rats with renovascular hypertension, H₂S inhibits the AngII-AT₁R signaling axis, downregulates myocardial AT₁R expression and AngII levels, while enhancing SOD activity and reducing ROS accumulation, creating a synergistic antioxidant-RAAS inhibitory effect (Liu et al., 2017). In isoproterenol-induced HF models, NaHS downregulates LTA4H expression and LTB4 production, inhibits mast cell degranulation, reduces renin release, improves diastolic function, and decreases collagen deposition (Liu et al., 2014). H₂S induces S-sulfhydration of MuRF1 at the Cys44 site, inhibiting its E3 ligase activity, reducing SERCA2a ubiquitination and degradation, thereby restoring SERCA2a function, lowering intracellular Ca^2+^, and enhancing contractile performance (Peng et al., 2022).

##### 3.2.3.5 Anti-inflammatory

The anti-inflammatory capacity of H₂S is evident across various pathological contexts. In hyperhomocysteinemia-induced myocardial injury, H₂S mitigates pathological progression through a four-pronged mechanism: (1) restoring CSE activity to lower plasma homocysteine levels, (2) reducing oxidative stress by decreasing lipid peroxidation product levels, (3) enhancing ROS scavenging through restored mitochondrial respiratory enzyme activity, and (4) inhibiting expression of ER stress markers (Chang et al., 2008). In doxorubicin-induced myocardial inflammation, H₂S directly suppresses p38 MAPK phosphorylation, blocking NF-κB nuclear translocation, while reducing ROS levels through antioxidative mechanisms to decrease pro-inflammatory cytokine transcription. In sepsis-induced myocardial injury models, H₂S downregulates TLR4 and NLRP3 protein expression, inhibits MyD88-NF-κB signaling activation to reduce cytokine secretion, and blocks NLRP3 inflammasome assembly and IL-1β processing (Xia et al., 2023).

In summary, H₂S exerts multifaceted cardioprotective effects: it regulates oxidative stress through direct ROS scavenging and enhanced antioxidant enzyme activity (e.g., MnSOD, Cu/Zn-SOD), preserving mitochondrial integrity. Improves myocardial metabolism by promoting fatty acid oxidation, glucose uptake, and hypoxia adaptation while reducing lipid accumulation and ER stress. Inhibits fibrosis by reducing collagen deposition, suppressing fibroblast activation, and counteracting the Warburg effect. Modulates neuroendocrine systems by inhibiting sympathetic-RAAS overactivation, enhancing neuroreflex pathways, and maintaining calcium homeostasis to improve cardiac function and remodeling. and demonstrates anti-inflammatory properties by reducing pro-inflammatory cytokines, inhibiting NLRP3 inflammasome activation, and blocking TLR4/NF-κB signaling in various pathological contexts (e.g., sepsis, hyperhomocysteinemia). These integrated actions establish H₂S as a crucial regulator of cardiovascular health, with therapeutic potential dependent on maintaining its balanced production and function.

#### 3.2.4 Indole metabolites

Human intestinal microorganisms primarily produce indole derivatives through the tryptophan metabolic pathway. The gut microbiota uses tryptophanase to degrade dietary tryptophan into indole, which is then further metabolized to indole acetic acid (IAA) and other derivatives. Dietary factors regulate this metabolic process: a high-fiber diet promotes beneficial bacteria to convert tryptophan into IPA, which exerts anti-inflammatory and antioxidant effects while inhibiting *Escherichia coli*-mediated indole overproduction. In contrast, low-fiber or high-protein diets may induce the accumulation of indole and toxic metabolites, thereby increasing susceptibility to intestinal inflammation and metabolic disorders.

Indole metabolites have garnered significant attention in cardiovascular disease research due to their bidirectional regulatory effects on cardiac function. These compounds offer protective effects but can also be toxic under certain pathological conditions. They inhibit radical-induced membrane lipid peroxidation and protein oxidation, thus preserving myocardial membrane integrity and functional stability. These actions reduce I/R-induced oxidative stress damage to cardiomyocytes and improve myocardial pump function (Andreadou et al., 2002). Indole metabolites scavenge excess ROS and free radicals generated during I/R, suppressing lipid peroxidation and protein oxidation (Broskova and Knezl, 2011). This mitigates structural damage to cardiomyocyte membranes while maintaining cellular functional integrity. Some indole derivatives exert synergistic protective effects through autophagy regulation. For example, the indole derivative IADB scavenges multiple ROS species and mimics SOD activity to enhance oxidative stress defense. It also upregulates LC3 protein expression, induces autophagosome formation, and enhances autophagic flux to alleviate 5-fluorouracil-induced cardiotoxicity (Bi et al., 2018).

Research on IPA has advanced significantly. IPA activates the NAD^+^ salvage pathway to optimize energy metabolism, regulates aryl hydrocarbon receptor (ARH) signaling to improve mitochondrial function, and enhances antioxidant capacity. Specifically, IPA activates the NAD^+^ salvage pathway to optimize energy metabolism. This increases nicotinamide and NAD^+^ bioavailability while inhibiting NNMT enzyme activity to reduce NAM methylation, thereby optimizing myocardial energy metabolism. Upon binding to AhR, IPA induces nuclear translocation and upregulates SIRT3 expression, enhancing mitochondrial antioxidant capacity and improving diastolic function (Wang et al., 2024). IPA accumulation demonstrates biphasic mitochondrial regulation: acute exposure (30 min) enhances maximal mitochondrial respiratory capacity, while chronic exposure (24 h) significantly inhibits complexes I/II/IV activity and fatty acid oxidation, reducing oxygen consumption rates (Gesper et al., 2021).

Furthermore, IPA suppresses the HDAC6/NOX2 pathway to decrease inflammatory cytokine production. It downregulates Bax/caspase-3 and upregulates Bcl-2 to inhibit apoptosis (Li et al., 2024), and activates the PI3K/AKT/GSK3*β* pathway to reduce apoptosis, improve contractile function, and alleviate *α*PD1-induced myocardial fibrosis and necrosis (Huang et al., 2025). IPA also activates AhR to inhibit NF-κB phosphorylation and NLRP3 inflammasome assembly, reducing IL-1β/TNF-α release (Zhang et al., 2024). Additionally, IPA upregulates ABCA1 to promote macrophage cholesterol efflux, thereby reducing foam cell formation (Xue et al., 2022). These integrated metabolic-signaling-anti-inflammatory networks provide novel therapeutic targets for intervention.

In summary, indole metabolites in cardiovascular disease exhibit conditional protective effects (e.g., inhibiting lipid/protein oxidation to preserve myocardial integrity, reduce ischemia/reperfusion injury, and enhance pump function). These integrated antioxidant, anti-apoptotic, anti-inflammatory, and metabolic properties position indoles as promising therapeutic targets for cardiovascular disease, emphasizing cellular protection and functional modulation.

#### 3.2.5 Polyphenols

Polyphenols, as plant-derived bioactive compounds, undergo gut microbiota-mediated metabolic transformation. Over 90% of colonic polyphenols are converted via enzymatic reactions (hydrolysis, dehydroxylation, reduction, cleavage) into small-molecule metabolites with enhanced bioavailability. These metabolites exert cardioprotective effects that are critically dependent on microbial metabolism (Mattera et al., 2017). The gut microbiota metabolizes large polyphenolic molecules into bioavailable metabolites through hydrolysis and dehydroxylation. In HF patients, gut dysbiosis reduces polyphenol metabolic efficiency, causing the accumulation of unmetabolized polyphenols in the intestine and diminished bioavailability. Pathogenic bacteria may generate toxic metabolites via aberrant metabolism, while reduced active metabolite production further impairs polyphenol-mediated regulatory effects.

Polyphenols exert antioxidant effects through dual mechanisms: direct scavenging of ROS and RNS via phenolic hydroxyl groups, and activation of the Keap1/Nrf2/ARE pathway to upregulate antioxidant enzymes. These effects are particularly pronounced in flavonoids, phenolic acids, and tannins (Mattera et al., 2017). As mitochondrial electron transport chain substrates, polyphenols enhance cytochrome c reduction, inhibit mPTP opening, and maintain membrane potential, thereby reducing ROS leakage from complexes I/III and preserving energy metabolism efficiency (Najjar and Feresin, 2021). Polyphenols suppress pro-inflammatory signaling by inhibiting NF-κB and MAPK/JNK phosphorylation. Specific compounds like quercetin regulate inflammatory cascades via peroxisome proliferator-activated receptor *γ* (PPARγ) activation, synergizing with antioxidant effects (Hedayati et al., 2023). Apoptosis inhibition involves multiple pathways: for instance, resveratrol can stabilize mitochondrial membrane potential, downregulate the Bax/Bcl-2 ratio, block cytochrome c release, activate the PI3K/Akt/mTOR pathway, upregulate the expression of anti-apoptotic Bcl-xL, and enhance cardiomyocyte survival (Najjar and Feresin, 2021). Metabolic regulation includes AMPK activation to promote fatty acid oxidation and improve cardiac energy metabolism, lipid profile modulation (LDL-C reduction/HDL-C elevation), and TGF-*β*/Smad pathway inhibition to reduce collagen deposition and myocardial remodeling. Calcium homeostasis is maintained through SERCA2a activation to enhance calcium reuptake and restore normal flux, while inhibiting Ca^2+^-dependent calcineurin/calpain/CaMKII activity to prevent calcium overload-induced remodeling (Hedayati et al., 2023).

In summary, the main functions of polyphenols include: antioxidant effects (ROS/RNS scavenging, upregulation of enzymes), anti-inflammatory effects (pathway inhibition, PPARγ modulation), anti-apoptotic mechanisms (mitochondrial stabilization, survival pathway activation), metabolic regulation (AMPK-driven fat oxidation, improvement of lipid profile, collagen reduction), and calcium homeostasis (SERCA2a activation, overload prevention). These properties highlight their cardiovascular therapeutic potential.

#### 3.2.6 Vitamins

Intestinal edema induces blood stasis and lymphatic drainage impairment, reducing fat emulsification and absorption capacity by 30–50%, thereby decreasing the absorption of fat-soluble vitamins. Concurrently, gut dysbiosis directly reduces intestinal synthesis of vitamins. Vitamin deficiency accelerates HF progression (McKeag et al., 2012), creating a vicious cycle: HF → intestinal disruption → vitamin deficiency → HF progression. This section focuses on the mechanisms and clinical value of vitamins B, C, D, and E in cardiovascular disease prevention and treatment, highlighting their efficacy differences and existing controversies.

Among hospitalized HF patients, 27% were vitamin B₂ deficient and 38% were vitamin B₆ deficient. Even after standard supplementation with B vitamins, there was no significant difference in deficiency rates between the supplementation and control groups (Keith et al., 2009), suggesting that HF patients may have abnormal utilization of vitamin supplements. RCTs further show that combined supplementation with folic acid, vitamin B₆, and vitamin B₁₂ does not reduce major cardiovascular event risk in vascular disease patients (Lonn et al., 2006), suggesting cautious evaluation of B vitamins’ anti-HF efficacy. Preclinical studies reveal sex-specific regulatory mechanisms: in female HF mice, the vitamin B complex (B3/B9/B12) optimizes energy supply through lipid metabolism remodeling while inhibiting Col1a1/Col3 gene expression and NLRP3 inflammasome activation, significantly reducing cardiopulmonary fibrosis and inflammatory cytokine levels. However, these sex differences lack sufficient clinical validation (David et al., 2025).

VC demonstrates more well-defined cardiovascular protective mechanisms. Elevated plasma VC levels correlate with reduced HF risk (Wannamethee et al., 2013). Clinical studies confirm that VC directly scavenges free radicals and lipid peroxidation products, mitigating oxidative stress damage. It also improves endothelium-dependent vasodilation (Ellis et al., 2000), with effects partially independent of antioxidant actions, possibly involving NO-mediated vasodilation or inflammation regulation. Long-term oral VC suppresses neutrophil superoxide anion production, further reducing inflammatory oxidative damage. Acute intravenous VC (2 g) enhances platelet responsiveness to NO donors and lowers oxidative stress levels (Ellis et al., 2001), indicating short-term therapeutic potential.

VD, functioning as both an endocrine and paracrine lipophilic vitamin, exhibits complex cardiovascular protective effects. Prospective cohort studies indicate that low 25(OH)D levels significantly increase all-cause mortality and HF readmission risks (Aleksova et al., 2020). RCTs reveal that high-dose VD₃ (4,000 IU/d for 1 year) fails to improve 6-min walk distance but enhances left ventricular ejection fraction and reduces ventricular volume, suggesting reversal of structural remodeling (Witte et al., 2016). In aging rat models, VD enhances SOD and catalase activity, reduces ROS accumulation and MDA production, improving redox balance. It also upregulates mitochondrial DNA-encoded respiratory chain subunits and biogenesis regulators, restoring mitochondrial biosynthesis and dynamic homeostasis (Shahidi et al., 2024). In D-galactose-induced aging models, VD reverses downregulation of mitochondrial autophagy mediators (Drp1/PINK1), inhibits cardiomyocyte apoptosis by modulating the Bcl2/Bax ratio, and maintains mitochondrial quality and cardiac structural stability (Shahidi et al., 2023). VD mediates direct regulation via widely distributed VD receptors in the cardiovascular system, balancing sympathetic/parasympathetic activity, correcting heart rate variability imbalance, and optimizing calcium channel kinetics for electrophysiological stability (Dogdus et al., 2019). Active VD supplementation reverses SERCA2a/PLB ratio decline and compensatory NCX upregulation caused by VD signaling defects, improving calcium handling and contractile dysfunction (Choudhury et al., 2014). In obese rat models, VD reduces cardiac TGF-β, NF-κB activity, and monocyte chemoattractant protein-1 concentration, alleviating inflammation and fibrosis (Ebrahimzadeh et al., 2022). It inhibits calcineurin A, ERK1/2, AKT, and TGF-β pathways, downregulates endoplasmic reticulum stress markers (GRP78/cATF6/CHOP), and upregulates SERCA2, thereby suppressing pressure overload-induced myocardial hypertrophy, fibrosis, oxidative stress, and apoptosis (Zhang et al., 2018).

VE faces significant clinical skepticism despite its theoretical membrane protection via lipid peroxidation chain reaction interruption. RCTs show that VE supplementation fails to significantly improve prognosis, functional markers, or quality of life in advanced HF patients (Keith et al., 2001).

In summary, vitamins B, C, D, and E exhibit distinct cardiovascular functional properties: B vitamins show limited efficacy in HF despite supplementation, with sex-specific metabolic regulation noted in preclinical models but lacking clinical validation. Vitamin C demonstrates robust antioxidant effects via free radical scavenging, lipid peroxidation inhibition, and endothelium-dependent vasodilation, with acute intravenous use showing short-term antioxidative benefits. Vitamin D exerts multifaceted protection through endocrine/paracrine actions: improving mitochondrial function, anti-fibrotic/anti-inflammatory effects, electrophysiological stabilization, and structural remodeling reversal. Vitamin E, despite theoretical membrane protection, lacks clinical efficacy in advanced HF prognosis. These findings highlight vitamin-specific therapeutic potentials and controversies in cardiovascular disease management, emphasizing the need for nuanced evaluation of supplementation strategies (Table 1).

**Table 1:** Metabolites appearing in the articles and the main mechanisms of action.

Metabolite	Derivative	Clinical efficacy	Mechanism of action
TMAO	Pro-inflammatory effect		Activation of the MAPK/NF-κB signaling pathway.
			m6A methylation regulation.
			Cascade effect of myocardial injury.
			Activation of the NLRP3 inflammasome.
			Vascular-myocardial inflammatory spread.
			Temporal dynamic bidirectional regulation.
	Pro-fibrotic effects		Activation of the TGF-β superfamily signaling pathway.
			ER stress and UPR.
			Fibroblast activation and collagen deposition.
			Epigenetic regulation.
	Oxidative stress		Disruption of the Ca^2+^–NO signaling axis.
			Induction of SIRT1-mediated mitochondrial dysfunction.
			Dysregulated mitochondrial metabolic reprogramming.
			Self-amplifying cascade of NOX/ROS/Nrf2/CES1 signaling.
			Paradoxical dual regulation by Nrf2.
	Metabolic dysregulation		Mitochondrial dysfunction and energy metabolic imbalance.
			Multi-pathway regulation of lipid metabolic disorders.
			Dose-dependent regulation under pathological conditions.
	Direct cardiac and electrophysiological effects		Direct cardiotoxicity (concentration/time-dependent).
			Dysregulation of calcium homeostasis and electrophysiological control.
			Sympathetic activation and electrophysiological instability.
			Dose- and context-dependent bidirectional effects.
PAA and its secondary derivatives	PAA	Oxidative stress and cellular senescence.	
		Inflammation- and fibrosis-driven processes.	
		Epigenetic dysregulation.	
		Energy metabolic inhibition.	
		Amplification of chronic vascular inflammation	
	PAGln	β2AR-mediated myocardial contractility and neurohormonal regulation.	
		Oxidative stress and mitochondrial damage.	
		Endothelial dysfunction and vascular inflammation.	
		Platelet activation and thrombotic risk.	
		Electrophysiological disturbances and structural remodeling.	
		Conditional protective effects.	
	Benzoic acid	Binding to mitochondrial thiols, exacerbating oxidative stress and disrupting energy metabolic balance.	
		Derivatives exhibit anti-inflammatory and antioxidant protective effects.	
		Derivatives possess antiplatelet and antithrombotic effects.	
LPS	Cardiac function and cardiac nerve damage		Calcium cycling and contractile dysfunction in cardiomyocytes.
			Neuroimmune crosstalk and sympathetic hyperactivation.
			Cross-pathway synergy and pathological amplification (TLR4–NF-κB, β1-AR–CaMKII–MAPK, PKC–MKP-1).
			Pathophysiological context-dependent regulation.
	Oxidative stress and autophagy imbalance		TLR4-dependent signaling axis dominates pathophysiology.
			Multidimensional regulation of oxidative stress.
			Bidirectional regulation of autophagy–mitophagy.
			Dysregulation of protein quality control.
			Ferroptosis and necrosis.
	Myocardial inflammatory response		Dual-pathway regulation of inflammatory cascades in cardiomyocytes.
			STING–IRF3–NLRP3 axis driving oxidative stress and pyroptosis.
			Local microenvironment disruption by NET release in atrial tissue.
			Regulation of metabolic enzymes and accumulation of toxic metabolites.
			NLRX1 ubiquitination and inflammatory amplification.
			Accelerated foam cell formation via macrophage internalization of immune complexes.
			mTOR–NF-κB axis driving the burst of inflammatory mediators.
			Dose-, disease-, and gender-specific manifestations.
Other cardiotoxic metabolites	IMP	Disruption of energy metabolism.	
	Cholesterol metabolites	Activation of inflammatory pathways.	
	TMAVA	Disruption of intestinal barrier function.	
	Indoxyl sulfate	Regulation of key signaling molecule.	
SCFAs	Acetate	Direct myocardial effects	Sympathetic regulation.
			Negative inotropic effect on cardiomyocytes.
		Energy metabolic regulation	Inhibition of fatty acid oxidation.
		Homeostatic regulation and anti-fibrotic effects	Neutralization of acidic microenvironment and inhibition of pathological calcium salt formation.
			Blockade of fibrotic signaling pathways.
		Indirect systemic protection	Gut microbiota remodeling.
			Metabolic and systemic regulation.
	Propionate	Metabolic bidirectional regulation and energy homeostasis	Promotion of glucose utilization and inhibition of fatty acid oxidation.
			Suppression of the TCA cycle.
		Alleviation of myocardial ischemic injury	Activation of GPR41 inhibits the Ang II–caveolin-1 axis, reducing I/R injury.
		Electrophysiological effects	Restoration of mitochondrial membrane potential improves contractile function.
		Chronic effects	In females, reduced contractility, promotion of diastolic dysfunction, and pathological myocardial remodeling.
	Butyrate	Cardiac function enhancement and stress protection	Non-β-adrenergic receptor pathways enhancing cardiac function.
		Anti-inflammatory and metabolic remodeling	Inhibition of NF-κB/STAT pathways.
		Promotion of mitochondrial biogenesis	Via the PGC-1α/TFAM pathway.
		Inhibition of cardiac fibroblast activation and collagen deposition	Suppression of TGF-β/Smad and CTGF pathways.
		Immunomodulatory effects	Activation of GPR41/43/109A receptors promoting Treg differentiation
BA and secondary BAs	BA	Energy substrate metabolic remodeling	Epigenetic modifications and metabolic enzyme regulation.
		Anti-inflammatory and metabolic protection	TGR5-dependent inhibition of NF-κB pathway and pro-inflammatory cytokines.
			FXR activation inhibits endothelin-1 transcription and Ang II signaling.
		Gut microbiota interaction	Secondary BAs generation reduces systemic inflammation.
	Secondary BAs	Improvement of cardiac functional parameters and structure.	
		Optimization of glucose metabolism.	
		Regulation of atrial structural remodeling in HF.	
		Enhancement of hemodynamic indices.	
H₂S	Oxidative stress regulation and mitochondrial protection		Direct ROS scavenging.
			Reduction of MDA levels.
			Enhanced endogenous antioxidant capacity.
			KEAP1 modification.
			Promotion of NRF2 nuclear translocation.
			Activation of the SLC7A11/GSH/GPx4 antioxidant axis.
			Increased AKT/FOXO3a phosphorylation.
			Alleviation of mitochondrial swelling.
			Regulation of α/β-myosin heavy chain ratio.
	Improved myocardial metabolism		Activation of the APN–AMPK signaling axis.
			Inhibition of glycolytic reprogramming.
			NaHS reverses inhibition of the PGC-1α–NRF1–TFAM pathway.
			Modulation of HIF-1α.
	Anti-fibrosis and autophagy regulation		Inhibition of atrial collagen deposition and cardiomyocyte apoptosis.
			Suppression of endoplasmic reticulum stress.
			Activation of PI3K/AKT/eNOS and eNOS–NO pathways.
			Activation of AMPK/mTOR and JAK2/STAT3 pathways.
			Inhibition of JNK/p38 MAPK and RIP1/RIP3/MLKL pathways.
			Na₂S donor inhibits sustained NLRP3 inflammasome activation.
			Downregulation of I/R-induced GRP78/CHOP/ATF6 expression.
	Neuroendocrine regulation		Decreased levels of renin, Ang II, and NE. Regulation of calcium homeostasis and recovery of contractile function.
	Anti-inflammatory		Inhibition of p38 MAPK phosphorylation and NF-κB nuclear translocation.
			Downregulation of TLR4/NLRP3 expression and inhibition of the MyD88–NF-κB signaling axis.
Indole metabolites	Antioxidant and membrane protection		Inhibition of lipid/protein oxidation.
			Protection of cardiomyocyte membrane integrity.
	Autophagy regulation		Enhancement of autophagic flux.
	Optimization of energy metabolism and mitochondrial function		Activation of the NAD^+^ pathway to optimize energy metabolism.
			AhR binding upregulates SIRT3 to enhance mitochondrial function.
			Biphasic regulation of mitochondrial respiratory capacity by IPA.
	Anti-inflammatory and anti-apoptotic pathway regulation		Inhibition of the HDAC6/NOX2 pathway.
			Regulation of apoptotic proteins.
			Activation of the PI3K/AKT/GSK3β pathway.
	Cholesterol metabolism regulation		Promotion of macrophage cholesterol efflux.
Polyphenols	Antioxidant		Direct scavenging of ROS/RNS.
			Activation of the Keap1/Nrf2/ARE pathway.
	Mitochondrial protection		Enhanced cytochrome c reduction.
			Inhibition of mPTP opening.
			Maintenance of membrane potential.
	Anti-inflammatory		Blockade of NF-κB and MAPK/JNK phosphorylation.
	Anti-apoptotic		Activation of the PI3K/Akt/mTOR pathway.
	Metabolic regulation		Reduction of LDL-C, elevation of HDL-C.
			Activation of AMPK.
			Inhibition of the TGF-β/Smad pathway.
	Calcium homeostasis maintenance		Activation of SERCA2a promoting calcium reuptake.
			Blockade of Ca^2+^-dependent calcineurin/calpain/CaMKII activity.
Vitamins	B	Anti-fibrotic and anti-inflammatory regulation	Suppression of Col1a1/Col3 gene expression and NLRP3.
	C	Antioxidant and vascular protection	NO-mediated reduction of oxidative stress
	D	Oxidative stress and mitochondrial regulation	Enhanced SOD/catalase activity and reduced ROS/MDA accumulation.
			Upregulation of COX1, COX2, and PGC-1α. Reversal of Drp1/PINK1 downregulation.
		Electrophysiology and calcium homeostasis	Optimization of calcium channel function.
		Anti-inflammatory and anti-fibrotic	Inhibition of TGF-β, NF-κB, ERK1/2, and AKT signaling pathways.
			Downregulation of ER stress markers.
	E	Membrane lipid protection	Interruption of lipid peroxidation chain reactions.

## 4 Intervention strategies, challenges, and future directions

Current intervention strategies based on the gut-heart axis theory demonstrate complex, multidimensional potential in comprehensive HF management, offering both efficacy and limitations. Probiotic therapy improves myocardial remodeling and cardiac function by modulating gut microbiota composition and function. For example, *Lactobacillus rhamnosus* GG and Bifidobacterium BB-12 strains protect the myocardium through mucosal biofilm formation, pathogen inhibition, maintenance of cellular energy metabolism, and reconstruction of immune homeostasis (Capurso, 2019; Merenstein et al., 2021). However, efficacy is constrained by strain specificity, dosage thresholds, and host genotype, with risks of microbiota resistance or immune hyperactivation in some patients. Prebiotic therapy (e.g., inulin, fructooligosaccharides) selectively corrects dysbiosis, positively influencing energy metabolism and myocardial mitochondrial biogenesis (Lai et al., 2020). However, excessive intake may induce gastrointestinal reactions (e.g., bloating, abnormal stools, lactose intolerance), and long-term efficacy depends on sustained intake and microbiome resilience. Fecal microbiota transplantation reverses HF-related dysbiosis by restoring gut microbiota *α*-diversity (Hu et al., 2019). However, donor safety (e.g., antimicrobial resistance genes, pathogen carriage, external factor sensitivity) requires stricter screening, and recipient immune rejection may affect durability (Hamamah et al., 2022). Metabolic toxin antagonists improve myocardial fibrosis by blocking harmful metabolite signaling pathways (Zhu et al., 2020). However, clinical application requires balancing target selectivity and off-target effects to avoid disrupting metabolic homeostasis. Dietary interventions (e.g., high-fiber, Mediterranean diets) optimize microbiota composition and reduce cardiovascular risk (Guasch-Ferré and Willett, 2021), but are hindered by patient adherence, racial/ethnic dietary differences, cooking practices, and regional economic factors. Traditional herbal ingredients, such as berberine, target LPS synthase in Gram-negative bacteria by downregulating lpxC and lpxD gene expression, thereby reducing LPS production and regulating metabolic pathways (including inhibiting TMAO synthesis and promoting SCFA secretion) while simultaneously repairing the intestinal barrier. However, the synergistic mechanisms of multi-component herbal compounds require further elucidation through systems biology approaches. Additionally, risk assessment of drug interactions faces challenges, including pharmacokinetic interactions, cytotoxicity, and interindividual variability (Zhang et al., 2025).

Future research should prioritize three key dimensions: individualized analysis, microbiota detection standardization, and intervention safety optimization. Individualized analysis focuses on multidrug resistance genes (e.g., Mrp2/Abcc2, Mdr1/Abcb1), which regulate probiotic colonization efficiency and inflammatory response intensity by modulating gut permeability and immune responses (Nakano et al., 2013). Drug-diet-microbiota interaction networks—such as proton pump inhibitor-induced gastric acid barrier disruption and high-fat diet-driven Firmicutes expansion—require multi-omics integration (metagenomics, metabolomics, and host genomics) to construct dynamic predictive models. This integration facilitates the transition from “one-size-fits-all” to personalized strategies (Mousa et al., 2023; Imhann et al., 2017). For microbiota detection standardization, multi-site intestinal mucosal sampling (rather than fecal samples) is recommended to capture spatial microbial diversity. Optimized sequencing depth (via primer design and library quality control) and bioinformatics pipelines (including UCHIME/UPARSE algorithms and high-quality reference genome databases) can enhance inter-study comparability (Guo et al., 2024). Standardized workflows minimize α-diversity measurement discrepancies across laboratories, establishing a reproducible framework for clinical decision-making. Intervention safety optimization involves leveraging engineered bacteria (with gene editing for precise metabolic pathway control) and multi-omics predictive models to forecast HF deterioration risk, providing molecular evidence for clinical alerts (Liu et al., 2022; Wang et al., 2025).

In clinical practice, the “personalized strategy” requires the development of a precise intervention framework centered on patient-specific biomarkers and dynamic microecological characteristics. For HF patients, individual differences can be identified through multi-omics integration (e.g., host genome sequencing, metagenome sequencing, and metabolomics). Specifically, patients with Mrp2/Abcc2 gene polymorphisms may exhibit increased intestinal permeability. Probiotics capable of enhancing intestinal barrier function (e.g., *Lactobacillus acidophilus*) should be preferentially selected, while drugs that may exacerbate intestinal barrier dysfunction (e.g., certain proton pump inhibitors) should be avoided. In cases where the Firmicutes/Bacteroidetes ratio in the intestinal flora is imbalanced, a high-fiber diet can be customized to promote the proliferation of SCFA-producing bacteria. This intervention can be combined with traditional Chinese medicine components such as berberine to inhibit LPS synthesis in Gram-negative bacteria. Additionally, by dynamically monitoring the composition, metabolite profile, and inflammatory markers (e.g., LPS and TMAO) in stool or intestinal mucosa samples, the treatment plan can be adjusted in real time. For example, when upregulated expression of drug-resistance genes (e.g., Mdr1/Abcb1) in the intestinal flora is detected, the risk of antibiotic use can be flagged in advance to prevent exacerbation of bacterial resistance. When Firmicutes expansion driven by a high-fat diet is identified, timely dietary interventions can be implemented to prevent further worsening of HF progression due to microbial imbalance. This dynamic adjustment strategy based on individualized biomarkers not only improves therapeutic efficacy but also minimizes drug interaction risks, thereby facilitating the transition from “empirical treatment” to “data-driven precision medicine” (Table 2).

**Table 2:** Strategies, potential mechanisms, and evidence.

Strategies	Potential mechanisms	Evidence
Probiotics/Prebiotics	① Competitively inhibit the colonization of pathogenic bacteria to maintain intestinal microbial balance. ② Promote the proliferation of beneficial bacteria and stimulate the production of protective metabolites. ③ Regulate intestinal pH to create a microenvironment conducive to eliminating pathogenic bacteria. ④ Activate intestinal lymphoid tissue to promote immunoglobulin secretion, repair mucosal damage, and restore barrier function. ⑤ Produce digestive enzymes that hydrolyze complex molecules (e.g., cellulose), enhance nutrient secretion/absorption, and improve drug bioavailability. ⑥ Certain probiotics modulate neurotransmitter secretion (e.g., dopamine, GABA) and regulate the neuroendocrine system. ⑦ Exert anti-inflammatory effects.	① Bifidobacterium and Lactobacillus inhibit pathogenic bacteria (e.g., *Escherichia coli*, *Helicobacter pylori*) by occupying intestinal adhesion sites and secreting lactic acid, bacteriocins, and other antimicrobial substances (Gryaznova et al., 2022). ② Prebiotic fibers enhance the growth of Bifidobacterium and Lactobacillus, optimize fat absorption, and promote the production of protective SCFAs (Green et al., 2020). ③ Lactulose, fructooligosaccharides, and inulin create a bactericidal microenvironment for enteric pathogens (e.g., *Clostridium perfringens*, *Escherichia coli*) by regulating intestinal pH (Green et al., 2020). ④ *Lactobacillus rhamnosus* GG and Bifidobacterium BB12 enhance mechanical mucosal barrier formation, upregulate tight junction proteins (e.g., ZO-1) in intestinal epithelial cells, and facilitate intestinal barrier repair (Ebrahimzadeh et al., 2022; Zhang et al., 2018). ⑤ Probiotic supplementation improves lactose absorption and utilization, maintains gut microbiota diversity, and modulates drug metabolism kinetics (Leis et al., 2020). ⑥ Microbiota restoration enhances intestinal function and stimulates enteric neurogenesis, increasing enteric glial cell and neuron numbers (Vicentini et al., 2021). ⑦ Specific probiotics/prebiotics exert anti-inflammatory effects by inhibiting TNF-α and increasing IL-10 (Roy and Dhaneshwar, 2023).
FMT	① Remodel gut microbiota to enhance beneficial bacteria abundance; ② Regulate immunity and inhibit inflammation to maintain intestinal barrier integrity; ③ Optimize metabolite profiles and metabolic pathways to enhance energy conversion efficiency.	① Introduce diverse gut microbiota from healthy donors (e.g., Firmicutes, Bacteroidetes, Bifidobacterium) to suppress pathogenic bacteria (e.g., Clostridioides difficile, *Escherichia coli*) via colonization resistance, thereby restoring gut microbiota diversity (Hou et al., 2025). ② Activate immune cells (e.g., dendritic cells, CD8 + T cells), inhibit pro-inflammatory cytokines, repair intestinal barrier integrity, enhance immune checkpoint inhibitor efficacy, and reduce immune-related adverse events (Lu et al., 2022). ③ Elevate SCFAs levels, enhance energy metabolism efficiency, and alleviate frailty symptoms in aged mice (Zhu et al., 2024).
Metabolic toxin antagonists	① Competitive exclusion of toxigenic bacteria to inhibit toxin production. ② Neutralize and adsorb toxins to enhance toxin excretion.	① Antibiotics suppress the proliferation of toxigenic bacteria (e.g., Clostridioides difficile, *Escherichia coli*) and reduce metabolic toxin production (e.g., LPS, ammonia, indole) (Lange et al., 2016). ② Adsorbents (e.g., Enterosgel) bind enterotoxins (e.g., endotoxin) to block absorption (Howell et al., 2019). antibodies (e.g., anti-LPS mAbs) neutralize toxin activity and reduce systemic circulation entry (Baumgartner, 1991).
Diet intervention	① Cultivate protective gut microbiota to optimize intestinal flora composition. ② Regulate inflammation, metabolism, and immune responses to maintain homeostasis. ③ Repair and restore intestinal barrier integrity to prevent pathogenic invasion.	① High-fiber diets promote the proliferation of beneficial bacteria (e.g., Bifidobacterium, Lactobacillus), while Mediterranean diets—rich in unsaturated fatty acids (olive oil, deep-sea fish)—increase anti-inflammatory bacteria (e.g., Akkermansia) (Gantenbein and Kanaka-Gantenbein, 2021). ② Low-calorie/low-fat diets and antioxidant-rich foods (vitamins C, E) reduce oxidative stress and inhibit pro-inflammatory cytokine release (Itsiopoulos et al., 2022). ③ Nutrients (e.g., glutamine, zinc) directly promote intestinal epithelial cell repair (Wang et al., 2024).
Traditional herbal remedies	① Directly remodel gut microbiota composition to promote commensal bacteria proliferation, suppress pathogenic bacteria growth, and enhance intestinal barrier integrity. ② The compound acts on multiple targets to enhance intestinal immune regulation and energy metabolism efficiency. ③ The compound acts on multiple targets to enhance intestinal immune regulation and energy metabolism efficiency.	① Herbal active compounds (e.g., polysaccharides, alkaloids, flavonoids) selectively stimulate commensal bacteria. Alkaloids (e.g., berberine, baicalin) directly inhibit pathogens. Yam mucilage restores mucosal barrier integrity, upregulates tight junction proteins (e.g., ZO-1), and prevents pathogenic invasion (Yue et al., 2022). ② Herbal ingredients exert synergistic effects to generate novel bioactive compounds, enhance therapeutic efficacy, improve bioavailability, and exert multi-target effects (e.g., NF-κB/PI3K-Akt pathways, SCFAs production) (Wang et al., 2024).

## 5 Conclusion

Through the integration of multi-dimensional evidence, this study systematically analyzes the interaction network between intestinal flora imbalance and HF. HF induces specific and multifaceted intestinal flora imbalances, including changes in microbial composition, intestinal barrier dysfunction, and microbial aging, all of which are closely linked to disease severity and prognosis. The effects of gut microbiota metabolites are highly context-dependent, with their bidirectional regulatory roles influenced by factors such as dose, disease stage, pathological background, and host characteristics (e.g., sex). While animal models have demonstrated the therapeutic potential of metabolites, clinical translation is still hindered by pathological complexity and limited understanding of the metabolic effects of current interventions. Personalized multi-omics strategies that integrate genomic, metabolomic, and clinical data are essential to overcoming these challenges. In the future, the gut-heart axis concept will revolutionize HF management through three major pathways: early diagnosis based on microbial metabolic signatures, real-time metabolic pathway intervention via dynamic regulatory platforms, and multi-omics risk stratification with efficacy monitoring to optimize prognosis evaluation. This paradigm is expected not only to transcend the limitations of traditional treatments but also to advance precision cardiology. It will redefine the entire-cycle intervention strategy—from prevention to palliative care—through targeted microbial engineering and metabolic reprogramming. Ultimately, it will accelerate clinical translation by developing platforms that mimic physiological conditions, exploring single-cell omics mechanisms, and implementing adaptive clinical designs, establishing the gut-heart axis as the cornerstone of precision medicine for HF.
